# Cardiac Pacemaker Cells Harness Stochastic Resonance to Ensure Fail-Safe Operation at Low Rates Bordering on Sinus Arrest

**DOI:** 10.1101/2024.12.19.629452

**Published:** 2026-05-04

**Authors:** Akihiro Okamura, Isabella K He, Alexander V Maltsev, Rostislav Bychkov, Syevda Tagirova, Michael Wang, Anna V Maltsev, Michael D Stern, Edward G Lakatta, Victor A Maltsev

**Affiliations:** 1 National Institute on Aging, NIH, Baltimore, MD 21224, USA; 2 School of Mathematics, Queen Mary University of London, London, UK

**Keywords:** Sinoatrial node, pacemaker, stochastic resonance, bradycardia, sinus arrest

## Abstract

**BACKGROUND:**

The sinoatrial node (SAN) is the primary pacemaker of the heart. Recent high-resolution imaging showed that synchronized action potentials (APs) exiting the SAN emerge from heterogeneous signals, including subthreshold signals in non-firing (dormant) cells. This raises a new question in cardiac biology: how do these signals contribute to heartbeat generation? Here, we tested the hypothesis that pacemaker cells harness stochastic resonance to ensure fail-safe operation, especially at low rates bordering on sinus arrest.

**METHODS:**

Membrane potential and Ca signals were measured using perforated-patch recordings in rabbit SAN cells exposed to sine-wave or white-noise currents. Additionally, we imaged Ca signals in intact mouse SAN tissue and performed multiscale model simulations at the subcellular, cellular, and tissue levels.

**RESULTS:**

In addition to classical synchronized Ca transients, SAN tissue exhibited heterogeneous local Ca signals of different kinetics. Noise currents, mimicking the heterogeneous natural cell environment, restored AP firing in dormant cells and substantially improved the rate and rhythm of those firing infrequently and irregularly. The benefit followed a bell-shaped curve: the performance improved but then declined, demonstrating a hallmark of stochastic resonance. Rhythmic AP generation in response to sine-wave currents of different frequencies defined a resonance spectrum in SAN cells, reflecting their ability to respond via stochastic resonance to specific frequency components embedded in noise. Cholinergic stimulation shifted the resonance spectrum and responses to noise toward lower frequencies across all amplitudes tested, rendering cells unresponsive to higher-frequency signals while enabling more effective processing of slower signals. Both the numerical models and simultaneous recordings of membrane potential and Ca dynamics demonstrated that stochastic resonance is amplified by coupled electrical and Ca signaling, enhancing AP generation at low noise levels. Adding noise currents to the cell and tissue models allowed firing under conditions where they otherwise would have stopped.

**CONCLUSIONS:**

SAN cells harness stochastic resonance amplified by coupled membrane-Ca signaling to ensure rhythmic heartbeat initiation, especially at low rates. This new signaling mechanism could help avoid sinus arrest when heart slows but noise increases, such as during parasympathetic stimulation, bradyarrhythmia, or aging.

## INTRODUCTION

The sinoatrial node (SAN) is a small region of heart tissue in the right atrium that generates rhythmic, spontaneous action potentials (APs) that pace cardiac contraction to meet the body’s demand for blood supply. Despite more than a century of research^1^, the origin of the heartbeat remains “still mysterious after all these years”^2^. This incomplete understanding of pacemaker mechanisms contributes, in part, to the fact that sinus node dysfunction, also known as sick sinus syndrome^3^, remains a major healthcare problem, especially in aged populations prone to bradyarrhythmia and life-threatening sinus arrest. Electronic pacemakers, currently used to treat sick sinus syndrome, impose lifestyle restrictions and can cause severe side effects.

Bradycardia occurs at the edge of normal SAN function and sinus arrest. What specific mechanisms maintain SAN function on this edge? The prevailing view of cardiac impulse initiation is that a small group of cells with the fastest AP firing rate then drives^4^ or entrains^5^ other SAN cells to deliver rhythmic impulses from the SAN exits. This view is consistent with earlier imaging of SAN tissue that showed concentric spread of excitation^6–8^. However, more recent imaging at single-cell resolution has revealed a new functional paradigm^9,10^. Excitation within the SAN center appears discontinuous and consists of functional cell communities (presumably signaling modules^11^) with distinct signaling patterns^9^. Some cells within the tissue generate no AP-induced transients^9,10^. Such non-firing cells (termed dormant cells^12^) represent a major population of cells enzymatically isolated from the SAN^12–15^. Dormant cells generate subthreshold noisy Ca and membrane potential oscillations, and many can be reversibly “awakened” to generate normal automaticity by β-adrenergic receptor stimulation^12,14–16^.

Thus, recent experimental data portray SAN pacemaking as an emergent property of a complex network of loosely connected, diversely firing and non-firing cells. The extreme structural heterogeneity of the SAN network, in which subthreshold irregular oscillations in some cells are mixed with AP firing in others, raises the possibility that random signal disturbances, often referred to as biological noise, could be an additional key factor in heart rate regulation^17,18^.

The role of biological noise in cardiac pacemaker function becomes even more apparent in light of experimental data suggesting that the heart’s pacemaker mimics brain cytoarchitecture and function^9,19,20^, and the cell cluster in the central SAN initiating the SAN impulse exhibits the most stochastic behavior^21^. Noise plays a fundamental role in information processing and affects all aspects of nervous-system function^22,23^. Because SAN tissue represents a complex network of interacting pacemaker cells and neuronal cells (the “little heart brain”^24^), it is reasonable to expect that in addition to channel noise within pacemaker cells and their membranes, noise sources found in neuronal networks, such as synaptic and sensory noise^22^, are also present in the SAN. For example, during cholinergic stimulation, individual local acetylcholine releases by nerve endings intensify and create disturbances in local signaling and AP-firing periodicity^25,26^. Additional noise sources influencing pacemaker cell function include ion-channel trafficking^27^ and connexin channels^28^.

Therefore, a frontier in cardiac biology is to understand the complex signal-processing mechanisms harbored within the heterogeneous SAN network that ultimately generate robust yet flexible cardiac impulses. Among those recently discussed^17,18,29–31^, one such signal-processing mechanism is stochastic resonance. Stochastic resonance is a phenomenon in which the addition of noise allows a subthreshold signal to become detectable by occasionally bringing the signal above threshold. Although stochastic resonance is recognized as a key information-processing mechanism in neuronal networks^32^, it has not been experimentally or theoretically demonstrated in SAN cells. Its presence and functional role in the SAN therefore remain unknown.

Each pacemaker cell exhibits intrinsic automaticity through diastolic depolarization generated by membrane currents coupled to periodic intracellular Ca signals within pacemaker cells^33^. Using combined experimental and theoretical approaches, the present study demonstrates that stochastic resonance is indeed a novel signal-processing mechanism in SAN cells that can support pacemaker function, especially at low AP firing rates both in basal state and during cholinergic receptor stimulation.

## METHODS

### Experimental methods and data availability

Detailed descriptions of experimental methods, materials, and statistical analyses, and numerical modeling are presented in the Supplemental Material. Experimental data associated with this study are available in Harvard Dataverse at https://doi.org/10.7910/DVN/PVEKM9.

## RESULTS

### Presence, patterns, and amplitude of Ca noise in intact SAN

**Figure 1.**
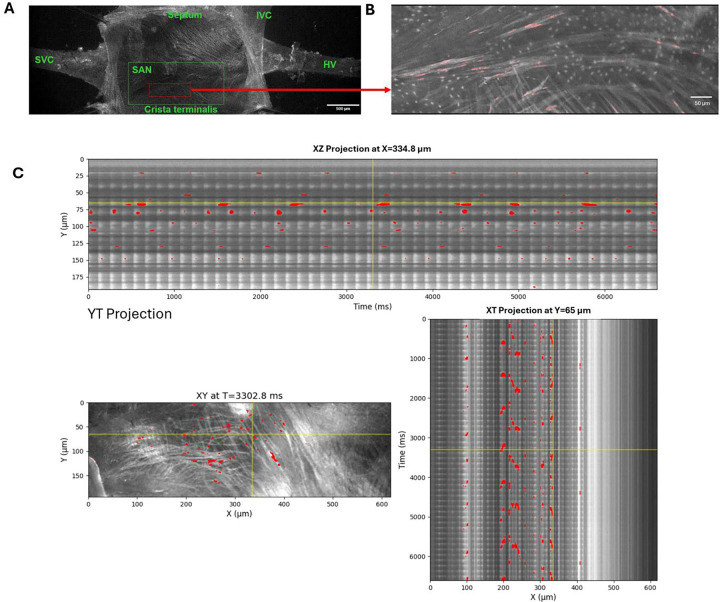
Presence of biological noise in intact SAN tissue. **A**, Low-zoom microscopic view of the entire mouse SAN preparation loaded with the Ca indicator Fluo-4, with anatomical benchmarks: crista terminalis, superior vena cava (SVC), hepatic vein (HV), and inferior vena ca**v**a (IVC). **B**, Higher-magnification view of a region of interest within the central SAN area. Scattered pink spots indicate background local Ca release (LCR) signals (Video S1). **C**, LCR signals (in red) detected in another SAN tissue preparation in a 2D image (XY) and in the corresponding XT and YT orthogonal projections (line-scan images along the yellow lines) to illustrate the spatiotemporal extent of the LCRs (Video S2). Our novel LCR-detection algorithm based on the PMD/Trefide/PCA signal-enhancement pipeline is described in detail in the Supplemental Material and Figure S1.

To characterize the noise environment relevant to stochastic resonance in SAN tissue, we performed high-resolution, single-cell-level Ca imaging in intact mouse SAN preparations using two complementary methods: high-speed sCMOS camera (n=6 mice, Figure 1, Videos S1-S5), and confocal microscopy (n=3 mice, Video S6).

Figure 1 and Video S1 show the high-speed camera recordings of Ca signals in the SAN tissue at a low zoom with key anatomical landmarks. Imaging at higher resolution revealed that, in addition to the “global” AP-induced Ca transients (APCTs) visible as periodic synchronized flashes throughout the tissue (Video S1, upper panel), substantial heterogeneous local Ca signals were present between and during the APCTs. To objectively separate these background local signals from global transients, we developed a novel PCA-based image analysis pipeline (detailed in Supplemental Methods and Figure S1). Subtraction of the principal component (global APCT signal) from the total Ca activity revealed the residual biological noise generated by individual cells as local Ca release (LCR) signals (Figure 1B, Video S1 and S2 with LCRs highlighted in pink). Spatiotemporal dynamics of LCR signals different from rhythmic APCTs are also clearly seen in orthogonal projections of the Ca image stacks (line-scanning images, Figure 1C).

We identified three distinct patterns of background noise signals with different kinetics: 1) intracellular cell-width Ca waves propagating along cell length generated rhythmic, low-frequency local signals (Videos S1-S3); 2) multiple smaller and faster LCR signals occurred within individual cells (Video S4); 3) incoherent firing, i.e. local AP-like signals out of synchrony with the global transient (Video S5). The amplitudes of noise signals were substantial, comparable in magnitude to APCTs recorded simultaneously. The presence of all three patterns was confirmed by confocal microscopy, which provided higher spatial resolution within individual cells (Video S6).

### Functional AP firing range of SAN cells revealed by sine-wave protocols

Following isolation, each pacemaker cell manifests its own preferred AP firing frequency, and some can be dormant (i.e., non-firing; Figure S2). Here we examined the range of frequencies at which a cell can generate rhythmic APs when it is entrained (one-to-one capture) by external periodic signals. To this end, we tested responses of 63 SAN cells to a sequence of external currents in the form of sine waves 2*a*sin(2π*F*t) of various frequ*e*ncies (F) from 0.5 to 6 Hz and amplitudes (a; current amplitude in one direction) from 10 to 50 pA (Figure S3, top). The resonance spectrum of a given cell at a given perturbation amplitude was defined as the frequency range within which AP firing matched the frequency of the applied sine wave (criterion: ±0.1 Hz).

**Figure 2.**
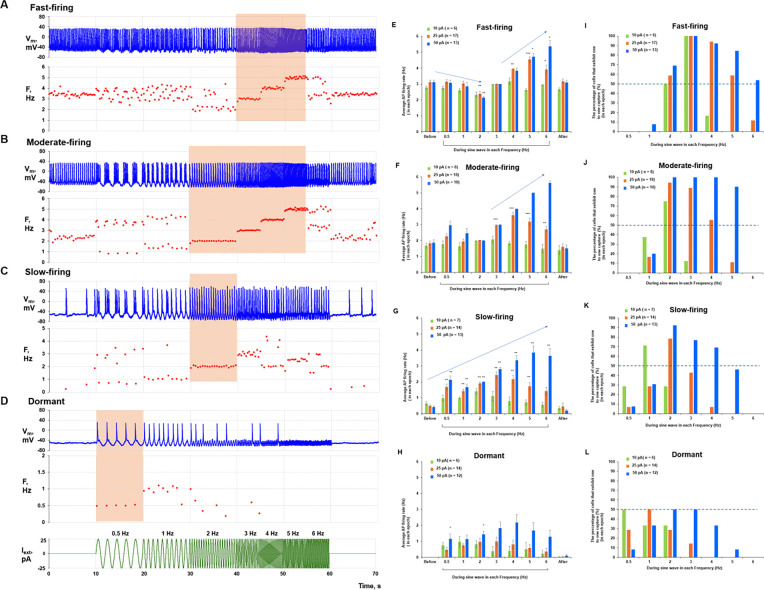
Resonance spectra of SAN cells revealed by sine-wave protocols. **A-D,** Representative examples of the effect of sine-wave currents (0.5 to 6 Hz, 25 pA; protocol shown at the bottom) on different cell populations. Original V_m_ traces and their corresponding intervalograms (in Hz) are shown. Resonance spectra are indicated by orange bands**. E-H**, Effects of sine-wave amplitude (color bars) on average AP firing rate (y-axis) at each sine-wave frequency (x-axis). Average AP firing rate in response to each sine-wave frequency was compared with that before sine-wave application using repeated-measures ANOVA or the Friedman test (*p < 0.05, **p < 0.01, ***p < 0.001). In addition, the Jonckheere-Terpstra trend test revealed significant trends in average rate change as wave frequency increased (arrows; tested separately and significant at p < 0.05 for 25 and 50 pA amplitudes). **I-L**, Percentage of cell**s** exhibiting one-to-one capture (y-axis) in response to each sine-wave frequency (x-axis) at each amplitude (colored bars). The horizontal dashed line indicates the 50% level and illustrates the stronger (broader) responsiveness of fast- and moderate-firing cells, especially at higher frequencies.

By applying a multitude of sine-wave protocols, we specifically measured the resonance spectra for four diverse cell populations: (i) fast-firing cells (>2.5 Hz, i.e. classical pacemakers); (ii) moderate-firing cells (1 to 2.5 Hz); (iii) slow-firing cells (< 1 Hz), and (iv) dormant cells firing no APs. Figure 2 shows examples of original recordings (panels A-D) and statistical analysis of the data (panels E-L). Our findings demonstrate that:

1. Larger-amplitude sine waves produced stronger effects, extending cell resonance spectra toward higher frequencies in a larger percentage of cells (blue bars, a = 50 pA, panels E-L).
2. All dormant cells began to fire APs as sine-wave amplitude increased.
3. The broadest resonance spectra were found for fast- and moderate-firing cells. The frequency range over which ≥50% of cells achieved one-to-one capture (dashed line in Figure 2, I-L) was the broadest in these cell populations (panels I and J) vs. that in slow-firing and dormant cells (panels K and L) at 25 and 50 pA amplitudes.
4. Inability of cells to follow low frequencies (0.5 and 1 Hz) signals led to burst firing: cells fired APs during the depolarizing part of the sine wave but fired no APs during the hyperpolarizing part (panels A-C). Conversely, dormant cells generated rhythmic firing at lower frequencies (as low as 0.5 Hz, panel D) but could not fire at higher frequencies (only 8% of cells could generate 5 Hz AP firing, panel L).
5. The resonance spectra shifted to lower frequencies as the intrinsic firing frequency of the cell population decreased. For example, at 50 pA amplitude the peak distribution was 3 Hz in fast-firing cells (blue bars, panel I), but only 2 Hz in slow-firing (blue bars, panel K).
6. Some dormant cells continued to fire after the sine-wave protocol ceased, displaying a “memory” effect (panel H, group “After”).
7. The resonance spectrum in each SAN cell is continuous. For example, cells maintained sine-wave capture as frequency increased in small 0.25-Hz steps, and capture occurred quickly, within 1 to 2 cycles (Figure S4).

### Stochastic resonance in SAN cells spontaneously firing APs

The diverse noise signals (Videos S1-S6) of varying kinetics described above provide the biological substrate in SAN tissue for stochastic resonance, the subject of the study. This Ca release noise in the SAN likely occurs in concert with other potential noise sources mentioned in Introduction, such as neuronal mediator release^22,25,26^, ion channel trafficking^27^ and connexin channels^28^. White noise has a constant power spectral density (possessing equal intensity across frequencies) and therefore, in our study, serves as a representative model for the heterogeneous, noisy environment within the SAN. We tested the effect of white noise using four amplitudes: 25, 31.25, 62.5, and 125 pA. Because our protocol was zero-mean, current disturbances occurred with equal probability in both depolarizing and hyperpolarizing directions (Figure S3, bottom). Thus, the cell’s response was not inherently intuitive, given the complex nonlinear interplay of numerous ion channels and transporters with distinct Ca and voltage sensitivities and kinetics.

**Figure 3.**
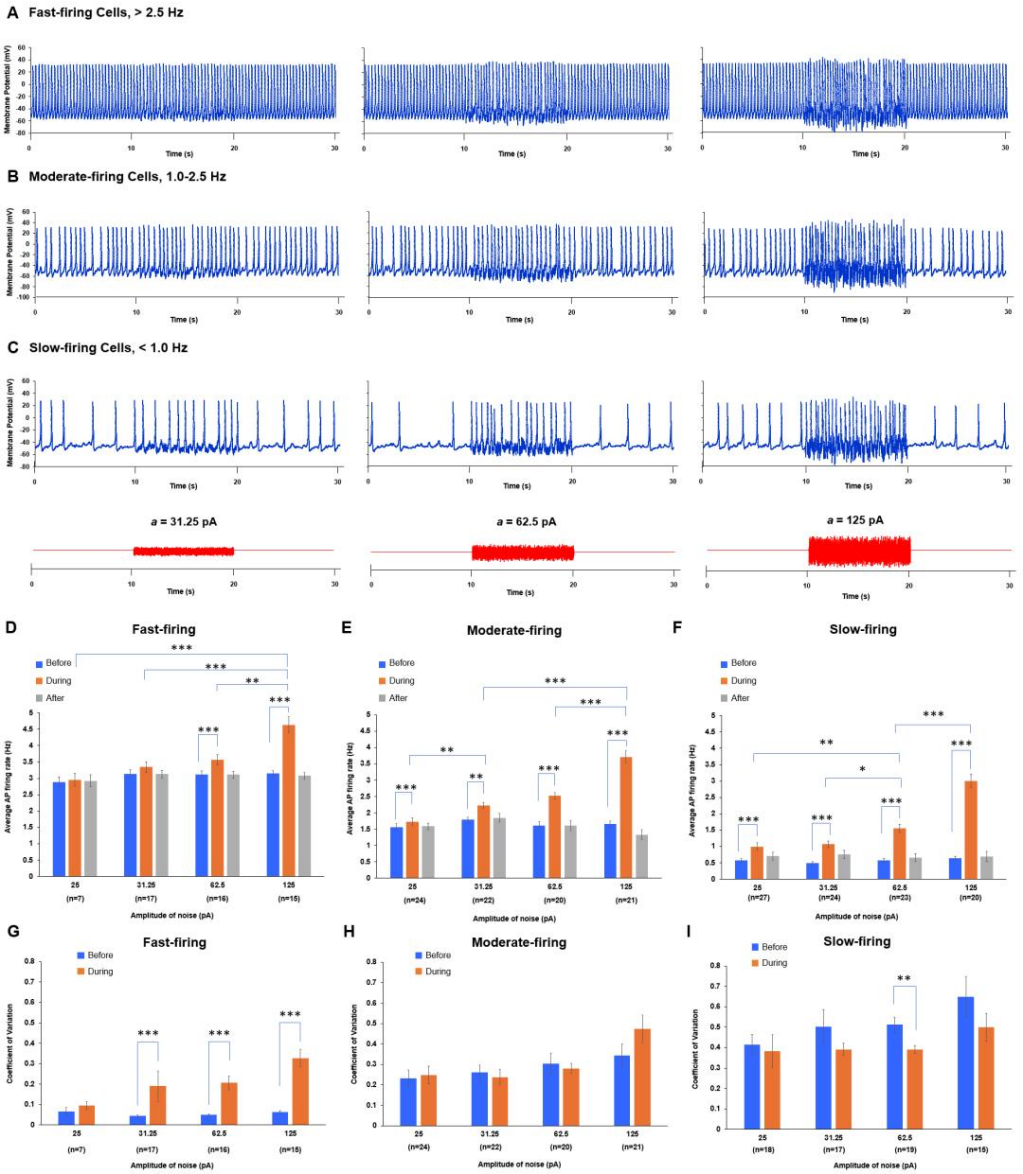
Stochastic resonance in SAN cell populations that spontaneously fired APs at different average rates. **A-C,** Examples of the effect of noise with different amplitudes (a) on AP firing in representative cells from each population. In all firing groups, noise increased AP firing rate, but it substantially perturbed the rhythmicity of fast-firing cells. **D-F**, Average AP firing rate was compared statistically before, during, and after noise application at each amplitude, and also among amplitudes (25, 31.25, 62.5, and 125 pA). In all cell populations, noise significantly increased AP firing rate (P < 0.05, repeated-measures ANOVA or Friedman test), except at 25 and 31.25 pA in the fast-firing group. There was no significant difference in AP firing rate between before noise application and after noise application was completed for any amplitude in any cell population. In the fast- and moderate-firing populations (**D and E**), 125 pA noise significantly increased the average AP firing rate compared with 25, 31.25, or 62.5 pA noise (p < 0.05, one-way ANOVA or Kruskal-Wallis test). In the slow-firing population (**F**), AP firing rate increased stepwise as noise amplitude increased. **G-I**, Similar statistical analyses were performed for the coefficient of variation (CV = SD/mean) of noise-induced AP firing. In the fast-firing SAN cell group (**G**), noise significantly decreased AP-firing rhythmicity (p < 0.05, paired t-test or Wilcox**o**n signed-rank test). In the moderate-firing group (**H**), there was no significant change in CV at any noise amplitude except 25 pA. In the slow-fi**r**ing group (**I**), noise significantly decreased CV at 25 and 62.5 pA. *p < 0.05, **p < 0.01, ***p < 0.001.

Panels A-C in Figure 3 show representative examples of the effect of noise on the firing frequency in each of 3 AP firing cell populations. Panels D-I show average AP firing frequencies and their coefficients of variation (defined as CV=SD/mean). An increase in CV indicates a decrease in rhythmicity and vice versa. Our findings are as follows:

1. For all populations of AP firing SAN cells and all tested noise amplitudes (except lower amplitudes of 25 and 31.25 pA in the fast-firing cells), the noise significantly increased the average AP firing frequency compared to intrinsic firing (orange bars vs. blue bars, Figure 3D-F). Furthermore, within each cell population, cells fired APs at significantly higher rates as the noise amplitude increased (statistical comparisons among orange bars in each cell population). Rarely, noise application decreased AP firing frequency at low noise amplitudes in some cells, but this fraction of cells (bars at x<0 in Figure S5) substantially decreased and disappeared as the noise amplitude increased.
2. Noise of a given amplitude had a more pronounced effect to increase firing frequency for slow-firing cells compared to moderate-firing and fast-firing cells (Figure 3 D-F).
3. In the fast-firing SAN cell population, noise decreased AP-firing rhythmicity. This was accompanied by a significant increase in the CV of AP firing frequency at noise amplitudes >25 pA (Figure 3G). An important and counterintuitive result was that noise application to slow-firing SAN cells improved AP-firing rhythmicity (CV decreased; Figure 3I).

### Stochastic resonance in dormant SAN cells

**Figure 4.**
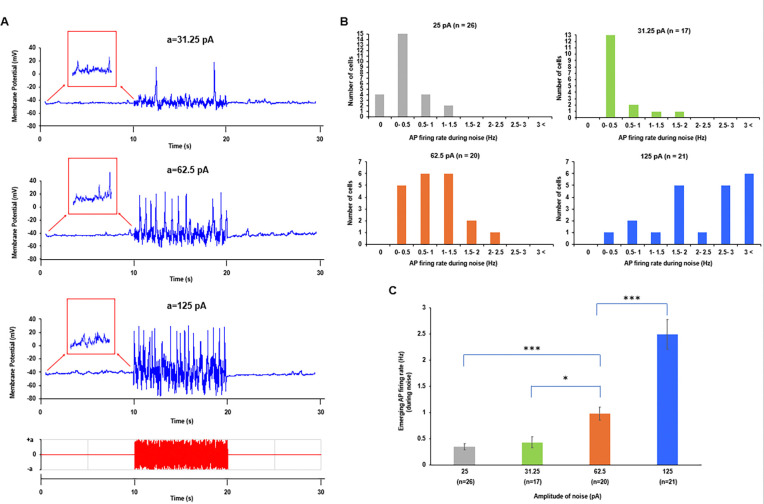
Stochastic resonance in dormant SAN cells. **A**, Representative examples of the effect of noise of different amplitudes on the AP firing rate. Before noise application dormant cells had subthreshold membrane potential oscillations (zoom-in, red squares) which were substantially increased in the presence of noise, reaching AP threshold and triggering AP firing, with the rate increasing as the noise amplitude increased. Moreover, the amplitude of noise-triggered APs substantially varied in each AP cycle, especially at larger amplitudes of noise. **B**, Distributions of the stochastic resonance effect for each noise amplitude tested. The noise awakened all dormant cells at noise amplitudes exceeding 25 pA. **C**, Average rate of AP firing (y-axis) caused by stochastic resonance increased as the noise amplitude increased (Kruskal-Wallis test). * p < 0.05, ** p < 0.01, *** p < 0.001.

Subthreshold signaling during the first 10 seconds of recording (red boxed insets, Figure 4A) increased markedly when noise was applied from 10 to 20 seconds, eventually surpassing the AP threshold and culminating in AP firing. Figure 4B shows the distribution of AP firing rates across all dormant cells tested at each noise amplitude. Noise induced AP firing in every cell tested at 31.25, 62.5, and 125 pA. Even the smallest tested amplitude (25 pA) induced AP firing in most cells (21 of 24). The mean frequency of noise-induced APs increased significantly as noise amplitude increased (Figure 4C), consistent with the sine-wave results: a broader resonance spectrum at larger amplitudes (Figure 2L) allows processing of higher-frequency signals via stochastic resonance. Importantly, although firing frequency increased at higher amplitudes, rhythmicity decreased, and extremely strong noise abolished this benefit, with membrane potential (V_m_) showing high-amplitude fluctuations instead of APs (Figure S6). This behavior is consistent with stochastic resonance, which characteristically shows peak benefit at an optimal noise intensity.

### Resonance spectrum and stochastic resonance during cholinergic receptor stimulation

**Figure 5.**
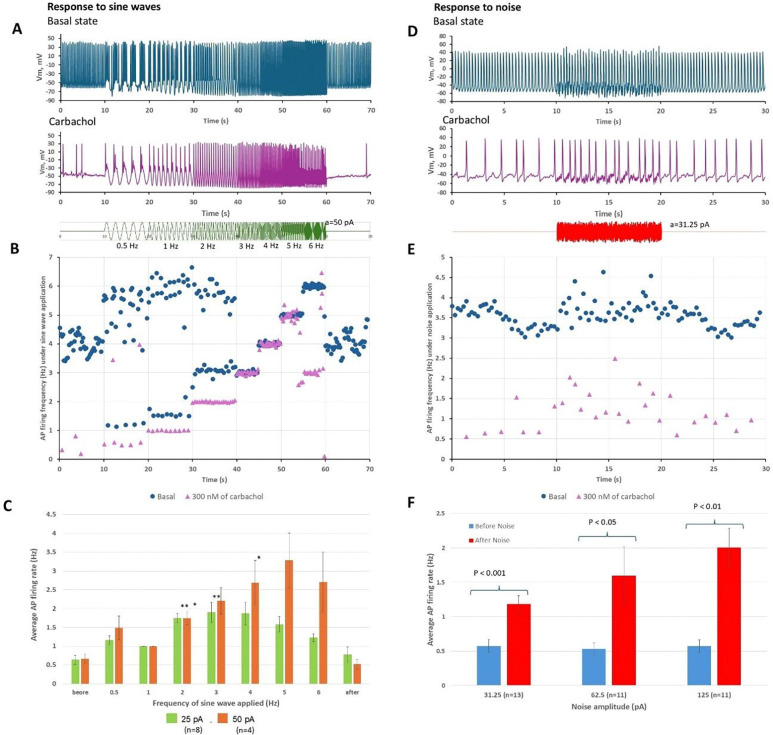
Resonance spectrum (functional range) and stochastic resonance in slow-firing cells during cholinergic receptor stimulation with carbachol. **A,** Original V_m_ recordings from the same cell in the basal state and in carbachol during the sine-wave protocol. The resonance spectrum shifted toward lower rates in the presence of carbachol. **B**, Intervalograms of the recordings in panel A. **C**, Statistical analysis of the effect of 25- and 50-pA sine waves on average AP firing rate (*p < 0.05, **p < 0.01, paired t-test for during-wave versus before-wave). **D**, Original V_m_ recordings from the same cell in the basal state and in carbachol during the noise protocol. Stochastic resonance increased AP firing rate in a representative slow-firing cell in the presence of carbachol. **E**, Corresponding intervalograms of the recordings in panel **D**. **F**, Statistical analysis of the effect of different noise amplitudes on averag**e** AP firing rate in cells under carbachol. The rate increased significantly at all amplitudes tested (P values are shown above the bars; paired t-test for during-noise versus before-noise).

To decrease AP firing rate and ultimately induce dormancy in some AP-firing cells, carbachol (Millipore-Sigma 212385-M, a synthetic analog of acetylcholine) was applied consecutively at 100, 300, and 1000 nM. In cells that continued to fire in carbachol, the resonance spectrum shifted toward lower frequencies, as shown by responses to sine-wave stimulation. Figure 5A shows an example: in the basal state the cell fired spontaneously at about 4 Hz, whereas under carbachol it fired at about 0.33 Hz (3 APs in 10 s). Under carbachol the cell no longer fired APs at 5 or 6 Hz but responded rhythmically at lower rates such as 1 and 2 Hz (intervalogram in Figure 5B). These lower rates were not achieved in the basal state. As larger sine-wave amplitudes were applied in the presence of carbachol, the cell achieved higher AP firing rates (Figure 5C).

Application of noise to firing cells under carbachol significantly increased their average AP firing rates (example in Figure 5D,E; population data in Figure 5F). As expected, an increase in firing rate is generally associated with lower AP rate variability, i.e. lower CV^34,35^. However, in our experiments a significant decrease in variability occurred only at a moderate (optimal) noise level. Thus, the benefit of rate improvement at stronger noise comes at the cost of greater rhythm disturbance, as large-amplitude noise introduces firing irregularity (Figure S7). This result is consistent with a hallmark of stochastic resonance: the presence of a maximum benefit at an optimal noise level.

**Figure 6.**
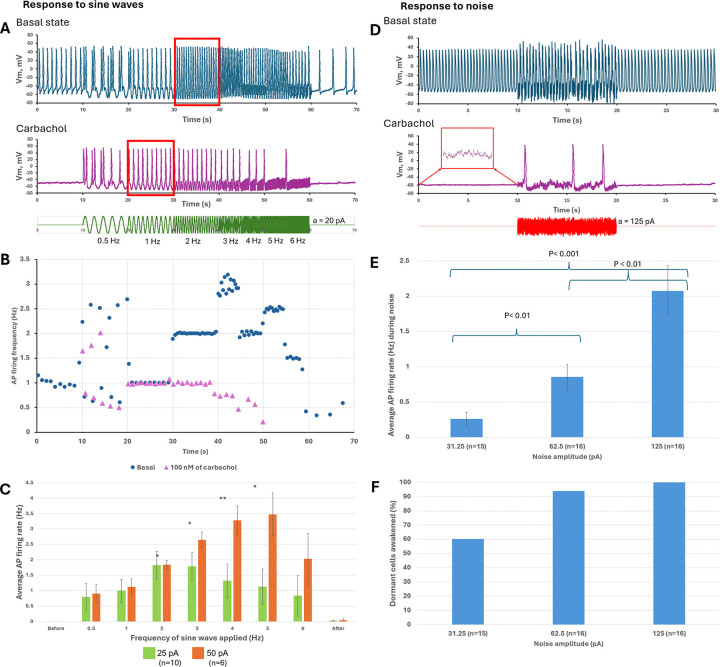
Resonance spectrum and stochastic resonance in dormant cells during cholinergic receptor stimulation with carbachol. **A,** Effects of a 20-pA sine-wave protocol in a representative cell in the basal state and after it became dormant in the presence of carbachol. The maximum resonance frequency shifted (red boxes) toward lower rates under carbachol (1 Hz) versus the basal state (2 Hz). **B**, intervalograms of recordings in **A**. **C**, Statistical analysis of the effect of sine waves of various frequencies and amplitudes (25 and 50 pA) on average AP firing rate (*p < 0.05, **p < 0.01, paired t-test for before-wave versus during-wave). **D**, Example of the noise effect on a dormant cell in the basal state and in the same cell during cholinergic receptor stimulation. The inset shows noisy intrinsic cell signals present under carbachol before noise was applied. **E**, Statistics for average AP firing frequency during noise at different amplitudes in the dormant-cell population. Statistical significance was determined by unequal-variance t-tests between pairs of different noise amplitudes (P values are indicated above each bar). **F**, Percentage of dormant cells awakened by a given noise amplitude.

Both sine-wave and noise protocols awakened dormant cells to generate APs (Figure 6). While a cell under carbachol began generating APs during sine-wave application, its maximum resonance frequency was reduced; for example, in panel A the maximum resonance frequency shifted from 2 Hz in the basal state to 1 Hz in carbachol (see red boxes in panel A and the intervalogram in panel B). A representative example of the noise effect is shown in panel D. Importantly, all dormant cells displayed noisy subthreshold V_m_ oscillation_s_ (red boxed inset in panel D), and these signals were amplified by noise to reach AP threshold via stochastic resonance. On average, larger sine-wave and noise amplitudes produced stronger effects, reflected in higher AP firing rates (panels C and E, respectively). Larger noise amplitudes also awakened a higher percentage of dormant cells (panel F). Thus, although cholinergic receptor stimulation shifts cell populations toward dormancy and slow firing, dormant cells can still be awakened to fire APs, and slow-firing cells can be induced to fire at higher rates, regardless of whether they occur naturally in the basal state or are induced by carbachol.

### Stochastic resonance is amplified by the coupled-clock system

**Figure 7.**
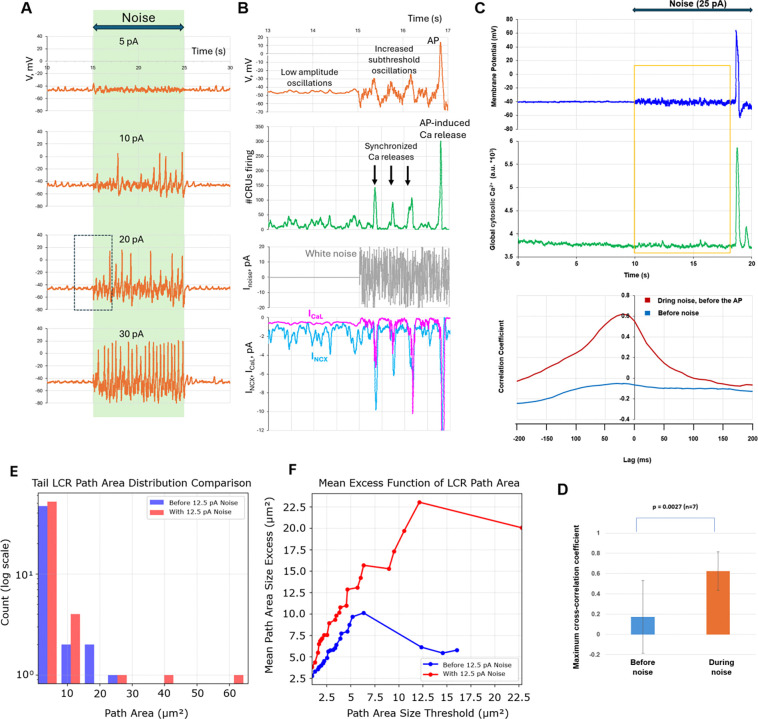
Stochastic resonance is amplified by the coupled-clock system in silico and in real cells. **A,** Responses of a dormant-cell model to noise of different amplitudes. **B**, Detailed comparison of cell signaling before and during 20 pA noise application before the first AP occurred (outlined by the box in panel A; see also Video S7). Synchronized CRU firing (green, downward black arrows), I_NCX_ (blue), I_CaL_ (magenta), and membrane-potential (V_m_) oscillations (orange) of increased amplitude, culminating in AP generation. **C**, Representative example of stochastic resonance in a representative dormant cell (see more examples in Figure S8) obtained in experimental simultaneous recordings of V_m_ and Ca signals (top panel), with the corresponding cross-correlation function for subthreshold V_m_ and Ca signaling before and during noise application (before the first AP fired). The time window chosen for cross-correlation analysis is shown by the orange square. **D,** Cross-correlation of subthreshold signals increased significantly during noise application, culminating in subsequent AP generation. **E**, Log-scale histogram revealing the architectural shift in LCR path areas between pre-noise (blue) and noise-present (red) states (cell #1 in Table S1). Noise exposure dramatically enriches the heavy-tail population of LCRs. **F**, To quantify heavy-tail behavior, large LCRs were further analyzed using mean-excess plots. Mean-excess analysis filters out LCRs above a given path-area threshold, calculates the average path area of the remaining larger LCRs, and compares this average with the original threshold. At higher cutoff levels, the average exceeds the threshold, confirming that large events become larger under noise conditions.

We used three state-of-the-art numerical models, representing pacemaker function at three different scales (subcellular, cellular, and tissue), to support our experimental findings and gain insight into specific mechanisms of stochastic resonance in SAN cells. At the subcellular level, we employed our recent agent-based SAN cell model^36^ (parameters in the Supplemental Material), in which individual Ca release units (CRUs) form an interactive functional network via Ca-induced Ca release that is coupled to a full set of membrane currents. Consistent with our experimental results (Figure 4A), white-noise currents awakened a dormant cell model to begin firing APs, with firing frequency increasing at stronger noise amplitudes (Figure 7A).

To gain insight into specific mechanisms of stochastic resonance in SAN cells, we compared subthreshold signaling before and during noise application in the dormant cell model. The amplitude of subthreshold V_m_ oscillations increased markedly during noise application because of coupled activation of CRUs, I_CaL_, and the Na/Ca exchanger current (I_NCX_), ultimately resulting in AP generation (Figure 7B). Ca releases became strongly synchronized in time and space and, as a result, increased in amplitude, with their peaks aligned with low-frequency inward-current components sensed and processed by the cell (resonance around 2 Hz; Video S7).

Experiments with simultaneous recording of V_m_ and Ca provided further evidence for subthreshold signaling amplification. The amplitude of the coupled subthreshold V_m_ and Ca fluctuations substantially increased in the presence of noise in the dormant cells (examples in Figures 7C, Figure S8, Video S8). To quantitatively demonstrate the increased coupling of Ca and V_m_ fluctuations, we examined their cross-correlations during noise (bottom sub-panels) and found that they significantly increased (Figure 7D). Importantly, to reveal genuine effects of subthreshold noise amplification, the cross-correlation was examined before the first AP was fired. Otherwise, the cross-correlation was always strong in AP firing cells independent of noise application, because each AP is strictly correlated with its attendant APCT (Figure S9).

To further explore how external noise amplifies cellular subthreshold signaling, we analyzed local Ca releases (LCRs) in dormant cells during simultaneous V_m_ and Ca recordings. Noise application in all analyzed cells (n=5) shifted LCR size distributions toward larger events quantified by a significant increase in L-kurtosis (mean 0.548 to 0.635; p<0.01; Table S1). LCR sizes were assessed as the entire Ca release propagation path area ^37^ reflecting CRU capability of recruiting neighboring CRU to fire via CICR mechanism. Under noise conditions, the LCRs exhibited a distinctly heavier tail (Figure 7E). Mean-excess analysis further confirmed that this shift reflected genuine enrichment of large-scale LCR events rather than uniform scaling (Figure 7F). This enrichment of large-scale LCRs reflects greater self-organization and synchronization of Ca release under noise.

### Stochastic resonance substantially expands the parametric space for AP firing in SAN cells

**Figure 8.**
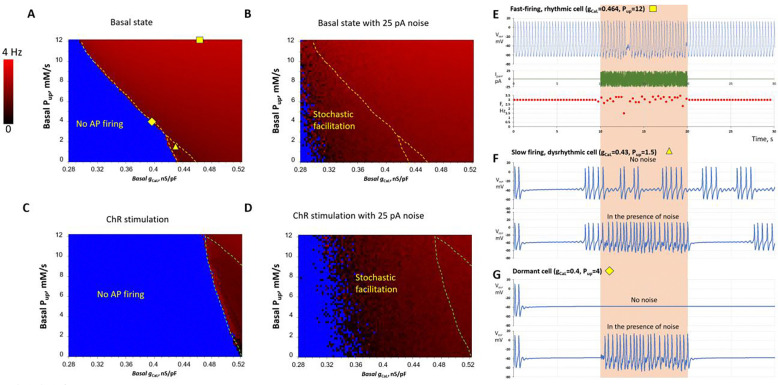
Insights from single-cell numerical modeling: stochastic resonance expands the parametric space of AP firing in the basal state and during cholinergic receptor stimulation. **A-D**, Each panel represents the result of numerical simulations of 5,917 (97 × 61 in xy) models with different coupled-clock parameters. I_CaL_ conductance (g_CaL_) was distributed evenly along the x-axis from 0.28 to 0.52 nS/pF in 0.0025-nS/pF increments. The sarcoplasmic reticulum Ca pumping rate (P_up_) was distributed evenly along the y-axis from 0 to 12 mM/s in 0.2-mM/s increments. Each pixel in the diagrams represents a single-cell model, with average AP firing frequency indicated by red shades. Blue areas indicate non-firing models. The blue area decreased substantially in the presence of noise. See Video S9 for V_m_ dynamics in all models. **E-G**, Representative examples of white-noise effects in different cell populations, with g_CaL_ and P_up_ values shown above the V_m_ traces in each panel. Noise disturbed the rhythmicity of AP firing in fast-firing cells but enhanced the function of slow-firing cells and awakened dormant cells to begin firing APs. The magenta double-headed arrow in panel A indicates the parameter range (g_CaL_=0.37 nS/pF, 0<P_up_<9.5 mM/s) used for the tissue-model simulations in Figure S12.

The “common-pool” Maltsev-Lakatta model^38^, which features faster computation, allowed us to test the effects of noise on coupled-clock function in a large number of derived cell models (n=23,668) representing different cell populations in the basal state and during cholinergic receptor stimulation. Experimental studies demonstrated that clock coupling determines the transition of a SAN cell from dormancy to rhythmic firing^14,16^. We therefore tested a wide variety of cell models with different basal-state firing activities generated by varying two key parameters of the coupled-clock system: I_CaL_ conductance (g_CaL_) and sarcoplasmic reticulum Ca pumping rate (P_up_). Variability in g_CaL_ reflects experimentally measured large-scale heterogeneity of I_CaL_ density^39–41^. Variability in P_up_ reflects a wide range of P_up_ inhibition levels such as arising from varying degree of phospholamban phosphorylation or pharmacological interventions. The diagram in Figure 8A shows the parametric space of firing cells in red shades, reflecting their average AP rates, and dormant cells in blue. Adding 25 pA noise awakened a major fraction of the dormant-cell population to fire APs, thereby extending AP firing (red area) toward the non-firing zone (blue area) of the parametric space in Figure 8B (see also Video S9). Representative examples of noise effects in the cell populations are shown in panels E-G: noise disturbed rhythmic firing in a fast-firing cell model, enhanced the rate and rhythm of a dysrhythmic slow-firing cell model, and awakened a dormant cell model to fire frequent APs, consistent with the experimental results (Figures 3A, 3C, and 4A). The stochastic resonance effect in awakening dormant cells was even more pronounced in the parametric space of SAN models during cholinergic receptor stimulation (Figure 8C,D; Video S9).

### Stochastic resonance prevents sinus arrest in a SAN tissue model

Parameters for the tissue model (Figure S12) were chosen so that most cells would lie in a non-firing zone, allowing the model to progress into sinus arrest after a few cycles over 6 s (Figure S12, Video S10). As previously demonstrated^42^, the transition to sinus arrest occurs via a phase transition in which dormant cells suppress the activity of all intrinsically firing cells. In the presence of noise, however, the SAN model continued to fire APs, clearly indicating that stochastic resonance prevents the phase transition of SAN tissue toward sinus arrest (Figure S12, Video S10).

## DISCUSSION

Recent high-resolution imaging studies portray a novel paradigm of SAN pacemaker function based on heterogeneous signaling within a brain-like cellular network^9,19^. The specific mechanisms driving pacemaking in such a complex network remain unknown. Noise plays a fundamental role in information processing and affects all aspects of nervous-system function^22,23^. One beneficial effect of noise in biological systems is stochastic resonance, which amplifies signaling in noisy environments and is used, for example, by neuronal networks for information processing^32^. Stochastic resonance models have also been conjectured for SAN function^17,18,29–31^. The present study provides the first direct experimental and theoretical evidence for the presence of stochastic resonance in SAN cells and its importance in pacemaker function.

### Specific mechanisms of stochastic resonance in SAN cells

While some SAN pacemaker cells beat spontaneously and are often called SAN myocytes, they share many properties with neuronal cells^43^. SAN cells express neuronal-type L-type Ca channels Ca_V_1.3^44^, neuronal-type Ca-activated adenylyl cyclases (AC1 and AC8)^45,46^, and the neuronal-type RyR3 Ca release channel^47^. They also share common properties with glutamatergic neurons^48^ and feature extended branches that support efficient long-range communication, as in neuronal networks. Pacemaker-cell interactions include pacemaker shift^49^, transitions to and from dormancy^10,12,14–16^, tonic entrainment^10^, and generation of complex signaling patterns, including rare firing and bursts of impulses^9,30^. Thus, SAN cells operate in a neuron-like manner: they detect, process, and respond to signals generated by other cells and, in turn, influence the operation of those cells.

The present study indicates that stochastic resonance is an important mechanism underlying these tasks. Depending on the signals processed via stochastic resonance, cells can shift their operating mode (i.e., firing modality^17^): a dormant cell can begin firing APs (Figure 4), a slow-firing cell can increase its firing rate (Figure 3), and cells can enter a burst-firing mode when low-frequency signals temporarily hyperpolarize the membrane (e.g., Figure 2A-C at 0.5 and 1 Hz). SAN cell AP firing can also depend on previous interactions, exhibiting a memory effect. For example, some dormant cells awakened by stochastic resonance continued firing APs even after noise removal (Figure 2H, group “After”; see also Figure S10). One possible mechanism is that cell activity becomes imprinted in altered states of sarcoplasmic reticulum Ca loading (short-term memory) or biochemical signaling (long-term memory), for example involving PKA, CaMKII, and adenylyl cyclases, which are critical for coupled-clock function in general^33^ and for restoration of AP firing in dormant cells in particular^14^. Thus, external signals can change the mode of SAN cell operation via stochastic resonance. The cell then responds by firing APs, sending signals to other cells in the collective SAN network for further information processing.

Signal processing and pacemaker-cell response depend on each cell’s resonance spectrum and its present operating status. The broadest resonance spectra were found in fast- and moderate-firing cells, whereas slow-firing and dormant cells exhibited narrower resonance spectra (orange bands in Figure 2A-D). Importantly, while resonance-spectrum width defines which frequency components can be utilized by a cell, the specific response to a complex perturbation (such as white noise in our experiments) depends on the cell’s operating point relative to AP threshold and on amplification of subthreshold V_m_-Ca oscillations. Therefore, dormant cells with a narrower low-frequency resonance spectrum can still undergo a qualitative transition from non-firing to firing (Figure 4), whereas among already firing cells the strongest rate facilitation was observed in slow-firing cells (Figure 3F).

Our numerical model simulations and simultaneous recordings of V_m_ and Ca revealed that stochastic resonance in SAN cells is not a simple electrical phenomenon: subthreshold signaling in SAN cells is strongly amplified by noise through coupled changes among local Ca releases, I_NCX_, V_m_, and I_CaL_ within the coupled-clock system (Figure 7, Figure S8, Videos S7 and S8). This powerful signal-amplification mechanism, involving positive-feedback interactions^50^, together with the broad resonance spectrum, makes stochastic resonance especially efficient in SAN cells for processing and responding to small subthreshold signals across broad frequency ranges.

In SAN tissue, such signals can be generated by other SAN cells operating at different frequencies and in different modes, by neuronal cells, and by other cell types (e.g., glial cells and S100B+/GFAP− interstitial cells^19^). All of these signals are, therefore, processed simultaneously in real time, analogous to analog computation in neurons^51^. Both stochastic resonance and the resonance spectrum of SAN cells are modulated by the autonomic system. For example, cholinergic receptor stimulation shifts them toward lower frequencies (Figures 5 and 6). As a result, the cells become unresponsive to higher-frequency signals but process lower-frequency signals more effectively.

### Stochastic resonance ensures fail-safe operation and prevents sinus arrest

In suppressing heart rate, the cholinergic receptor stimulation decreases the strength of diastolic pacemaker signals, with stronger stimulation culminating in SAN sinus arrest. In terms of evolution, the low-heart rate regime is extremely important for survival as it allows for energy conservation until most needed, such as during the fight-or-flight response. This regime must be balanced by powerful and robust signal processing mechanisms that prevent sinus arrest. Stochastic resonance can be one of them, because bradycardic cholinergic receptor stimulation simultaneously increases overall biological noise in the SAN cells and tissue due to: 1) increased LCR stochasticity in SAN cells^52^; 2) an increased number of dormant cells^10^, that generate stochastic LCRs^12^; and 3) intensification of individual local releases of acetylcholine by nerve endings^25,26^. We show, in turn, that noise can stimulate dormant cells to fire APs (Figure 4), including those that become dormant during cholinergic receptor stimulation, and substantially enhance the rate and rhythm of cells firing infrequent, dysrhythmic APs (Figure 3). Furthermore, our numerical simulations indicate that these noise-mediated enhancements likely operate in a large fraction of SAN cells (Figure 8A-D and Video S9) and prevent sinus arrest in SAN tissue (Figure S12 and Video S10). In contrast, deterministic, limit-cycle models of SAN cells (lacking stochastic components) do not provide such robust function^53^.

Organisms harness stochasticity to generate a wide range of possible solutions to environmental challenges and to make optimal choices for future action^54^. In this sense, our study suggests that SAN cells and the SAN cellular network can employ stochastic resonance together with criticality-related behavior^2,55^ to expand the space of possible solutions, and thus the repertoire of complex responses, that help navigate future firing pattern and avoid AP cessation at low AP firing rates.

With respect to the fast-firing cell population, the effect of noise in these cells does not neatly fit the classic definition of stochastic resonance. These cells generate their own frequent rhythmic signals during each diastolic depolarization and reach AP threshold without further external amplification or prompting, whereas noise application leads to their dysrhythmic firing (Figure 3A,G). We also observed decreases in firing rate in some cells exposed to small and moderate noise amplitudes (Figure S5, bars at x<0; example in Figure S11), consistent with inverse stochastic resonance, in which noise suppresses oscillation frequency^56^. Such noise-induced suppression adds further complexity to the ways in which noise can regulate cell AP firing and, ultimately, heart rate. Thus, while noise is a universal modulator of SAN cell behavior, its role as a beneficial stochastic-resonance mechanism is most prominent at lower firing rates.

A recent conceptual model of stochastic resonance in the SAN^17,30^ has proposed that “noisy” cells with sporadic APs and subthreshold voltage fluctuations in the inferior SAN stabilize rhythm and prevent pauses, that is, prevent burst firing in the superior SAN. Our single-cell experiments partly support this hypothesis: if a single cell generates irregular bursts of AP firing, application of an optimal external noise can indeed eliminate the pauses (Figure 3B, middle panel). Furthermore, noise can promptly restore normal, rhythmic pacemaker function in populations of slow-firing cells (Figure 3C and 3F) and dormant cells (Figure 4). Our numerical model simulations demonstrate that stochastic resonance can improve pacemaker function at multiple scales across a wide range of key model parameters (Figures 7 and 8).

Previous studies in SAN tissue have shown that the central portion of the node actually beats slower than portions closer to the cristae terminalis when these pieces of the node are separated from each other^57,58^. Additionally, SAN pieces extracted near the left atrium were nonfunctioning until adrenaline or acetylcholine were applied. Taken together, these reports and our findings suggest that distinct clusters within the sinus node, characterized by varying pacemaking activity, may possess different sensitivities to stochastic resonance when integrated into a functional tissue network. For example, the SAN central pacemaking site may rely on stochastic resonance to maintain a faster beating rate. When isolated from the surrounding tissue, its rate may slow because it no longer receives noise input from neighboring cells. This reasoning is consistent with a recent experimental report that the cell cluster in the central SAN initiating the SAN impulse exhibits the most stochastic behavior^21^.

### New approaches to study the new pacemaker paradigm

In the classical paradigm of concentric excitation propagation within SAN tissue, emphasis was placed on parameters governing cell automaticity (I_f_), excitability (I_CaL_ and I_Na)_, and tissue conduction (connexins). In the emerging paradigm of information processing within the complex SAN cellular network^9,19^, additional parameters become important, including cell clusters forming brain-like small-world modular networks with efficient signal processing^11,59^; cell resonance spectra (Figure 2), which are critical for stochastic resonance efficiency; diversely firing cell populations^12,14,15^ (we studied four here); diversity of cell sensitivity to autonomic modulation^60^; phase-like transitions in which dormant cells prevail and the SAN abruptly falls into sinus arrest^42^; and locally released mediators that orchestrate cell activity and tune network operation. An extreme example of the importance of heterogeneity and clustering was recently demonstrated by two dormant cell populations that fired no APs in isolation but generated rhythmic APs when connected within a functional network^31^.

### Study limitations, future studies, and importance for aging and translational research

Although our experiments were performed in rabbit SAN cells, these cells share fundamental pacemaker mechanisms with human SAN cells ^16^, including coupled-clock operation, similar ion-channel expression, and heterogeneous cell populations featuring dormant cells. Critically, biological noise and background Ca signals analogous to those characterized here in rabbit SAN cells have been directly observed in human SAN tissue (Video 5 in Bychkov et al.^9^), supporting the translational relevance of our findings. The main species difference relevant to stochastic resonance is the mean heart rate, which would be expected to shift the resonance spectrum toward lower frequencies in humans. Further studies in human SAN cells will clarify how specific differences in ion channel densities, cell coupling, and autonomic innervation affect stochastic resonance characteristics.

Future studies in cells isolated from specific anatomical regions, as in^30^, and in new models such as brain-like small-world SAN models^59^, will examine how stochastic resonance operates within and among pacemaker cells, functional modules^9,11,30,31,59,61^, and other cell types in the SAN. Because stochastic resonance reflects a general ability of ion-channel systems to detect and amplify small signals in noisy environments^62^, and because threshold-dependent firing in noisy heterogeneous tissues is not unique to the SAN, future studies should also examine its role in other cardiac tissues, such as atrial and ventricular muscle, latent pacemakers in the atria, the AV node, and Purkinje fibers that generate escape rhythms when SAN function fails.

These questions are especially relevant to aging. Disorder within biological systems tends to increase with aging across scales, suggesting that heterogeneity and noise in SAN also increase with age. Aging-associated fibrosis, tissue fragmentation, and cell decoupling are likely to reduce transmission of noise between cells, but they can also increase the amplitude of local noise within individual cells by confining current fluctuations that would otherwise spread electrotonically to neighboring cells. Intermediate coupling supports SAN tissue beating through interactions between AP-firing and dormant cells^42,63^, whereas noise can be beneficial under partial decoupling, for example when ion-channel opening-closing noise increases SAN AP firing in Cx30-deficient mice^64^. Older SAN cells appear less able to generate and process higher-frequency signals^65^, consistent with the shift of the resonance spectrum and stochastic resonance toward lower frequencies that we observed during cholinergic receptor stimulation (Figures 5 and 6). Thus, stochastic resonance likely becomes more important in supporting function of the aged SAN at low rates, partially compensating for the reduced intrinsic heart rate and helping prevent sinus arrest.

Sick sinus syndrome associated with bradyarrhythmia and sinus arrest remains a major healthcare problem, especially as the elderly population grows^3^. Furthermore, sinus node dysfunction affects up to one in five patients with atrial fibrillation^66^ and is also associated with severe bradyarrhythmia in heart failure^67^, which can lead to sudden cardiac death. Our findings may help develop therapeutic strategies that, rather than relying solely on direct pacing, restore or substitute for complex cellular signals that are lost or degraded with aging, thereby treating bradyarrhythmia and preventing sinus arrest.

Another potential avenue is the development of biological pacemakers. Although this idea was proposed more than two decades ago^68,69^, progress has been limited, in part, by incomplete understanding of the fundamental principles governing SAN cellular-network operation^2^. The emerging paradigm of cardiac pacemaker function^2,9,10,17–19,29–31^, with stochastic resonance as a key component, suggests that bioengineering strategies should be revisited on a new conceptual basis to create biological pacemakers that more faithfully mimic the native heart.

## Supplementary Material

Supplement 1

Supplement 2

Supplement 3

Supplement 4

Supplement 5

Supplement 6

Supplement 7

Supplement 8

Supplement 9

Supplement 10

Supplement 11

Supplemental Material

Supplemental Methods

Methods for LCR signal detection in isolated SAN cells

Algorithm for LCR signal detection in SAN tissue

Computer code and specific model parameters used in the present study

Supplemental Figures S1–S12

Table S1

Videos S1-S10

Legends for Videos

Glossary of Key Terms
