# Supplementary material for: Cardiac Pacemaker Cells Harness Stochastic Resonance to Ensure Fail-Safe Operation at Low Rates Bordering on Sinus Arrest
Source: bioRxiv. 2026 May 4:2024.12.19.629452. Preprint. [Version 2] doi: 10.1101/2024.12.19.629452 (PMC13174630; doi:10.1101/2024.12.19.629452)
Supplement: Supplement 11 [file NIHPP2024.12.19.629452v2-supplement-11.pdf]

bioRxiv preprint doi: https://doi.org/10.1101/2024.12.19.629452; this version posted May 4, 2026. The copyright holder for this preprint (which was not certified by peer review) is the author/funder. This article is a US Government work. It is not subject to copyright under 17 USC 105 and is also made available for use under a CC0 license.

Supplemental Methods

Rabbit SAN cell isolation

SAN cells were isolated from male rabbits as previously described[70], in accordance with NIH guidelines for the care and use of animals (protocol #457-LCS-2024). New Zealand White rabbits (Charles River Laboratories, USA) weighing 2.8–3.2 kg were anesthetized with sodium pentobarbital (50–90 mg/kg). The heart was removed quickly and placed in a solution containing (in mM): 130 NaCl, 24 $NaHCO_3$, 1.2 NaH_2PO_4, 1.0 $MgCl_2$, 1.8 $CaCl_2$, 4.0 KCl, and 5.6 glucose, equilibrated w$_i$th 95% O_2 / 5% CO2 (pH 7.4 at 35°C). The SAN region was cut into small strips (~1.0 mm wide) perpendicular to the crista terminalis and excised. The final SAN preparation, consisting of SAN strips attached to a small portion of the crista terminalis, was washed twice in nominally Ca-free solution containing (in mM): 140 NaCl, 5.4 KCl, 0.5 $MgCl_2$, 0.33 NaH_2PO_4, 5 HEPES, and 5.5 glucose (pH 6.9) and incubated on a shaker at 35°C for 30 min in the same solution supplemented with elastase type IV (0.6 mg/mL; Sigma Chemical Co.), collagenase type 2 (0.8 mg/mL; Worthington, NJ, USA), protease XIV (0.12 mg/mL; Sigma Chemical Co.), and 0.1% bovine serum albumin (Sigma Chemical Co.). The SAN preparation was then placed in modified Kraftbruhe (KB) solution containing (in mM): 70 potassium glutamate, 30 KCl, 10 KH_2PO_4, 1 $MgCl_2$, 20 taurine, 10 glucose, 0.3 EGTA, and 10 HEPES (titrated to pH 7.4 with KOH), and kept at 4°C for 1 h in KB solution containing 50 mg/mL polyvinylpyrrolidone (PVP; Sigma Chemical Co.). Finally, cells were dispersed from the SAN preparation by gentle pipetting in KB solution and stored at 4°C.

bioRxiv preprint doi: https://doi.org/10.1101/2024.12.19.629452; this version posted May 4, 2026. The copyright holder for this preprint (which was not certified by peer review) is the author/funder. This article is a US Government work. It is not subject to copyright under 17 USC 105 and is also made available for use under a CC0 license.

Electrophysiology and Ca signal imaging

Perforated patch-clamp recording was used in current-clamp mode to measure V_m and apply external membrane currents of different waveforms. Membrane patch perforation was achieved using β-escin (50 μM) added to the patch-pipette solution, as described elsewhere [71]. The patch-pipette solution contained (in mM): 120 K-gluconate, 5 NaCl, 5 Mg-ATP, 5 HEPES, 20 KCl, and 3 Na_2ATP (pH adjusted to 7.2 with KOH). Axopatch 200B, DIGIDATA 1440, and pCLAMP software (Molecular Devices, USA) were used for data acquisition with a sampling interval of 0.1 ms.

Ca imaging was performed as previously described [14]. Cells were loaded with 5 μM Fluo-4AM for 20 min at room temperature before measurement. Ca signals were imaged with a 2D sCMOS camera (PCO edge 4.2) with a 13.2-mm square sensor and 2048 x 2048 pixel resolution. Images were acquired at 100 frames/s, which required use of only part of the sensor (1280 x 1280 pixels). The recording camera was mounted on Zeiss Axiovert 100 inverted microscopes (Carl Zeiss, Inc., Germany) with a 40x oil-immersion lens and a CoolLED pE-300-W fluorescence excitation light source (CoolLED Ltd., Andover, UK). Fluo-4 fluorescence was excited at 470/40 nm and emission was collected at 525/50 nm using Zeiss filter set 38 HE.

Measurements of V_m separately or simultaneously with Ca were performed at 36°±0.1°C (500 μl chamber volume). Temperature was controlled by an Analog TC2BIP 2/3Ch bipolar temperature controller from CellMicroControls (Norfolk, VA, USA). This heated both the glass bottom of the perfusion chamber and the solution entering the chamber (via a pre-heater). The physiological (bathing) solution contained in mM: NaCl 140; KCl 5.4; $MgCl_2$ 2; HEPES 5; $CaCl_2$ 1.8; pH 7.3 (adjusted with NaOH). In order to perform simultaneous recording of Ca signals and membrane potential, we programmed the PCO camera software to generate a TTL

bioRxiv preprint doi: https://doi.org/10.1101/2024.12.19.629452; this version posted May 4, 2026. The copyright holder for this preprint (which was not certified by peer review) is the author/funder. This article is a US Government work. It is not subject to copyright under 17 USC 105 and is also made available for use under a CC0 license.

signal when imaging started. This signal triggered V_m recording via pCLAMP software. Video-recordings of intracellular Ca signals were analyzed by ImageJ program.

Cell populations tested in the study

We tested four cell populations based on intrinsic activity: (i) fast-firing cells (>2.5 Hz, i.e., classical pacemakers); (ii) moderate-firing cells (1 to 2.5 Hz); (iii) slow-firing cells (<1 Hz); and (iv) dormant cells firing no APs. Notably, dormant cells had morphology similar to that of classical pacemaker cells, being mainly spindle-shaped (Figure S2). Dormant cells were either (i) naturally dormant, that is, generating no APs at zero current clamp; (ii) rendered dormant by application of carbachol, a synthetic analog of ACh; or (iii) rendered dormant by passage of a hyperpolarizing current simulating electrotonic interactions with neighboring cells, such as the influence of other dormant cells [10] or cells near the atrial tissue surrounding the SAN.

Mouse heart isolation

The mouse heart was dissected as previously described [9]. Experimental protocols were approved by the Animal Care and Use Committee of the National Institutes of Health (protocol #034-LCS-2019). The heart was removed quickly and placed in standard Tyrode solution containing (in mM): 130 NaCl, 24 $NaHCO_3$, 1.2 NaH_2PO_4, 1.0 $MgCl_2$, 1.8 $CaCl_2$, 4.0 KCl, and 5.6 glucose, equilibrated with 95% O2 / 5% CO2 (pH 7.4 at 35.5°C).

bioRxiv preprint doi: https://doi.org/10.1101/2024.12.19.629452; this version posted May 4, 2026. The copyright holder for this preprint (which was not certified by peer review) is the author/funder. This article is a US Government work. It is not subject to copyright under 17 USC 105 and is also made available for use under a CC0 license.

Preparation of mouse SAN tissue and Ca signal imaging by high-speed camera

Preparation of mouse SAN tissue has been described in detail in [9]. In short, the whole heart was pinned to a silicon platform under a surgical microscope in order to excise the right and left atria. A 10-ml tissue bath was perfused with standard solution at a rate of 10 ml/min. After removal of the ventricles, the right atrium was opened to expose the crista terminalis, the inter-caval area, and the inter-atrial septum. The preparation was not trimmed, leaving SAN region together with surrounding atria and superior and inferior vena cava (SVC and IVC) intact. The SAN preparation was pinned to the silicon bottom of the experimental chamber by small stainless-steel pins with the endocardial side exposed. Care was taken to provide the minimal amount of stretch required to flatten the SAN tissue. After mounting, the preparation was superfused with solution maintained at a temperature of 36±0.3°C.

We used the imaging system described previously [9] to assess intracellular Ca dynamics within individual cells across the intact mouse SAN. In brief, we used a stationary fixed-stage upright microscope (AxioExaminer D1 equipped with a zoom tube [0.5–4x], Carl Zeiss Microscopy LLC) and a PCO edge 4.2 camera featuring a scientific complementary metal-oxide semiconductor (sCMOS) sensor with high spatial and temporal resolution. The experimental chamber containing the SAN preparation was placed on a platform (Sutter Instruments) mounted on a pressurized air table (Newport).

The SAN preparation was incubated with a membrane-permeable Ca indicator Fluo-4AM (10μM) for 1.5 hours. The excitation light was generated by CoolLED pE-300ultra. The excitation light was reflected to the SAN preparation by a dichroic mirror with a central wavelength of 498 nm, and the emitted fluorescence signal was collected through a 530±20 nm filter (Semrock, USA). The fluorescence image of the SAN preparation was projected by air or water lenses onto the

bioRxiv preprint doi: https://doi.org/10.1101/2024.12.19.629452; this version posted May 4, 2026. The copyright holder for this preprint (which was not certified by peer review) is the author/funder. This article is a US Government work. It is not subject to copyright under 17 USC 105 and is also made available for use under a CC0 license.

sCMOS camera sensor. To prevent interference of tissue motion during recordings from SAN tissue, we decoupled electrical excitation and mechanical contraction in some preparations by inhibiting the formation of the Ca-sensitive regulatory complexes within sarcomeres using 10 µM cytochalasin B [72].

Preparation of mouse SAN tissue and Ca signal imaging by confocal microscopy

The heart was transferred to oxygenated Tyrode solution (35 ± 0.5°C) containing (in mM) 140 NaCl, 5.4 KCl, 1.2 KH_2PO_4, 1.0 $MgCl_2$, 1.8 $CaCl_2$, 5.55 glucose, and 5 HEPES, with pH adjusted to 7.4 with NaOH. It was pinned onto a silicone dish, the right atrium was opened, and the SAN region, including the superior and inferior venae cavae (SVC and IVC), was dissected and pinned with the endocardial side facing upward on a silicone ring. Calbryte 520 AM (20 µM) was added to the SAN preparation in oxygenated Tyrode solution, and the preparation was left for ~2 h at room temperature. After 2 h, the preparation was washed and transferred to the experimental chamber, where the silicone ring was turned upside down (SAN endocardial side facing downward) for data acquisition (35 ± 0.5°C). Ca signals were recorded in Tyrode solution using a 10x/0.3 objective with an LSM 510 META confocal microscope (Carl Zeiss) in frame mode with 488-nm excitation. The pinhole was ~4.2 Airy units; the spatial scale was ~0.21–0.48 µm; the stack size was ~108–245 µm; scan zoom was ~3.7–8.3; and laser power was ~5%–15%.

Statistics

Values are expressed as mean ± standard error. Normality was tested with the Shapiro-Wilk test. Normally distributed continuous variables were compared using the paired t-test. One-way

bioRxiv preprint doi: https://doi.org/10.1101/2024.12.19.629452; this version posted May 4, 2026. The copyright holder for this preprint (which was not certified by peer review) is the author/funder. This article is a US Government work. It is not subject to copyright under 17 USC 105 and is also made available for use under a CC0 license.

analysis of variance (ANOVA) with Bonferroni correction, repeated-measures ANOVA when appropriate, and the Wilcoxon signed-rank, Kruskal-Wallis, or Friedman tests for skewed data were used as appropriate. Trends in average AP firing rate during sine-wave application were assessed with the Jonckheere-Terpstra trend test. A value of $p < 0.05$ was considered statistically significant.

Methods for LCR signal detection in isolated SAN cells

LCR analysis was performed using a custom Python pipeline that implements and extends the computational framework for LCR detection described in our previous work[37,73]. To ensure valid paired comparisons of LCR characteristics, equal-length time windows were extracted before and during noise application.

When TIFF image stacks are first loaded, the signal intensity is globally normalized by min-max scaling. Then to delineate the cell cytoplasm from the background, a binary cell mask is generated by creating a maximum-intensity projection over time and thresholding the resulting image at its mean intensity. The largest resulting connected component is retained as the final cell mask. Global fluorescence decay and drift are removed by subtracting the mean intensity within the cell mask from each frame and any negative differences are set to zero to produce a detrended image stack.

To emphasize the rising phase of Ca signals, frame-to-frame differences of the detrended signal were computed, and negative differences were again set to zero. Unlike our previous single-threshold approach[37], which applies a one-pass cutoff based on the standard deviation (σ) of fluorescence intensity within the cell region to baseline-subtracted fluorescence, we applied a

bioRxiv preprint doi: https://doi.org/10.1101/2024.12.19.629452; this version posted May 4, 2026. The copyright holder for this preprint (which was not certified by peer review) is the author/funder. This article is a US Government work. It is not subject to copyright under 17 USC 105 and is also made available for use under a CC0 license.

novel two-stage hysteresis thresholding procedure to these temporal differences to yield more robust, noise-resilient detection of true Ca release events. First, an initial seed threshold at sd_seed x σ (sd_seed = 1.0) identified core release areas; next, those cores were grown by including connected pixels above a lower growth threshold at sd_grow x σ (sd_grow = 0.5). Finally, candidate regions underwent connected-component labeling, size filtering, and morphological operations (closing, opening, and hole filling) to produce the final binary LCR mask for each frame.

Finally, LCR events were reconstructed into full spatiotemporal trajectories by linking regions across consecutive frames on the basis of maximal pixel overlap, a process that resolves LCR collisions and logs births, branches, and deaths, as described previously[37]. From these complete trajectories, per-event metrics were extracted, including path area, lifetime, and initiation/termination times, for subsequent statistical analysis. Our Jupyter Notebook for the full LCR analysis is available on GitHub (https://github.com/alexmaltsev/SANC-LCR-Analysis).

Algorithm for background signal detection in SAN tissue

To better observe and analyze LCRs and other intrinsic noise patterns in SAN tissue, we developed a signal-enhancement pipeline centered on Penalized Matrix Decomposition (PMD), implemented through the Python package Trefide [74]. Originally designed to extract neuronal Ca and voltage signals from brain-imaging data, this algorithm is well suited to analysis of complex spatiotemporal Ca dynamics in cardiac tissue, including the stochastic signals relevant to pacemaker function.

bioRxiv preprint doi: https://doi.org/10.1101/2024.12.19.629452; this version posted May 4, 2026. The copyright holder for this preprint (which was not certified by peer review) is the author/funder. This article is a US Government work. It is not subject to copyright under 17 USC 105 and is also made available for use under a CC0 license.

The pipeline begins with a custom automated preprocessing sequence to delineate the active tissue area in the raw TIFF movie (Figure S1A). First, the video's contrast is enhanced using histogram normalization, where the intensity range is set by saturating 0.35% of the brightest and dimmest pixels. Next, a Gaussian Mixture Model (GMM) is fit to the maximum intensity projection to automatically define a tight bounding box around the SAN tissue. The raw video is then cropped to this box, and any remaining pixels whose maximum intensity across all frames fall below the mean maximum intensity of the field of view are masked and set to zero.

Following preprocessing, the prepared imaging data undergoes signal enhancement through PMD as implemented in Trefide (Figure S1B). Within each spatial patch (40×40 pixels), the algorithm identifies and extracts distinct signal activity patterns through a constrained optimization process that iteratively finds spatiotemporal components maximizing data variance while enforcing spatial coherence and temporal smoothness, capturing up to 50 separate signal sources per patch. In SAN tissue, these spatiotemporal components correspond to synchronized Ca transients within the tissue (which are later removed in further processing), propagating Ca waves, or localized Ca release events.

The decomposition ensures biological plausibility by applying mathematical constraints: a spatial penalty (Total Variation) maintains connected regions rather than scattered pixels, while a temporal penalty (Trend Filtering) preserves smooth Ca dynamics with sharp upstrokes characteristic of Ca events. To prevent the extraction of spurious patterns from measurement noise, the algorithm automatically terminates when it fails to identify statistically significant signal patterns in three consecutive attempts.

After processing all patches independently, Trefide reconstructs a complete enhanced movie by combining the extracted Ca signals through weighted averaging in overlapping

bioRxiv preprint doi: https://doi.org/10.1101/2024.12.19.629452; this version posted May 4, 2026. The copyright holder for this preprint (which was not certified by peer review) is the author/funder. This article is a US Government work. It is not subject to copyright under 17 USC 105 and is also made available for use under a CC0 license.

regions, where signals present in multiple patches are blended based on their proximity to patch centers. This results in dramatically improved visualization of Ca activity, revealing previously obscured Ca dynamics including the stochastic Ca release patterns essential for understanding how SAN cells harness biological noise to ensure rhythmic heartbeat generation. Our Jupyter Notebook for the full SAN analysis is published on GitHub. (https://github.com/alexmaltsev/SAN-Analysis)

Following signal enhancement, Principal Component Analysis (PCA) removes the dominant global Ca transient from underlying local signals (Figure S1C). This approach draws from random matrix theory applications to cardiac tissue, as demonstrated by Norris & Maltsev [11], who showed that the first principal component of SAN Ca imaging data captures the collective, action potential (AP)-driven Ca wave propagating across the tissue. Our implementation follows a similar algorithm: it reshapes the signal-improved 3D movie into a 2D matrix (time × pixels), performs Singular Value Decomposition (SVD) to compute the first principal component, and subtracts it from the post-Trefide processed data. The resulting residual movie preserves local Ca signaling patterns while removing the dominant global transient.

Finally, to create an objective final mask of LCRs and other intrinsic noise patterns in SAN tissue, we used statistical methods from Extreme Value Theory (EVT) to derive a nonarbitrary, data-driven threshold for separating events from background fluctuations (Figure S1D). After subtraction of the global PCA component, most pixel residuals collapsed around zero, leaving approximately the remaining <10% of variance [11] split between small background fluctuations and occasional large-amplitude Ca transients which, by virtue of their magnitude and limited spatial footprint, form the extreme right-hand tail of the residual-intensity distribution. Thus, an optimal gray threshold could be computed to determine where heavy-tail events begin.

bioRxiv preprint doi: https://doi.org/10.1101/2024.12.19.629452; this version posted May 4, 2026. The copyright holder for this preprint (which was not certified by peer review) is the author/funder. This article is a US Government work. It is not subject to copyright under 17 USC 105 and is also made available for use under a CC0 license.

To determine this threshold, the algorithm fit a Generalized Pareto Distribution (GPD) to data values exceeding 100 different thresholds spanning quantiles from the 50th to 99th percentile. For computational efficiency, fitting proceeded only when at least 50 pixels exceeded a given threshold, and the data were downsampled to a maximum of 10,000 points. The key insight lies in analyzing the stability of the GPD shape parameter (ξ) as a function of threshold: the optimal threshold occurs where this parameter interpolates to zero. Applying this EVT-derived threshold to clip the residual movie effectively isolates LCRs as the remaining signals for subsequent analysis (Figure 1 and Figure S1E; see also Videos S1 and S2).

Computer code and specific model parameters used in the present study

A key part of our study was numerical model simulations that supported our experimental results and provided further insights into how specifically stochastic resonance contributes to pacemaker function at three scales: (i) the subcellular scale, using Ca-Release-Unit (CRU)-based models[36]; (ii) cellular scale, using common pool models[38]; and (iii) SAN tissue scale, using a 2D grid multi-cellular model[42].

Code availability

We employed three numerical models at different scales:

1. Single SAN cell model at the subcellular level was our CRU-based (agent-based) model [36]. Its computer code (Delphi language) is freely available as supplementary data to our original publication[36]. Code for more recent model versions is also freely available on GitHub:

bioRxiv preprint doi: https://doi.org/10.1101/2024.12.19.629452; this version posted May 4, 2026. The copyright holder for this preprint (which was not certified by peer review) is the author/funder. This article is a US Government work. It is not subject to copyright under 17 USC 105 and is also made available for use under a CC0 license.

https://github.com/victoramaltsev/CRU-based-SANC-model (with various CRU sizes [75]) and https://github.com/victoramaltsev/RyR-network-SANC-model (with individual RyRs [76]).

2. Single SAN cell model at the cellular level (common-pool model) was the Maltsev-Lakatta model [38]. The computer code for the original model is freely available in CellML format (maltsev_2009_paper.cellml) at http://models.cellml.org/workspace/maltsev_2009 and can be executed using the Cellular Open Resource (COR) software developed at the University of Oxford by Garny et al.[77] (for recent development of COR, see http://www.opencor.ws).

3. The SAN tissue model used here is an adapted version[42] of the tissue model originally developed by Campana. The model code in CUDA C is freely available in Campana's PhD thesis, Appendix A[78]: https://amslaurea.unibo.it/8596/1/campana_chiara_tesi.pdf

Parameters of noise

The noise current I_{noise} was generated as a sequence of random numbers within a range of [-a, a] with an interval of 4 ms (Figure S3). Intrinsic membrane current I_m together with I_{noise} drives V_m change: $dV_m/dt = -(I_m+I_{noise})/C_m$, where C_m is cell membrane capacitance in the tested models and in real cells.

bioRxiv preprint doi: https://doi.org/10.1101/2024.12.19.629452; this version posted May 4, 2026. The copyright holder for this preprint (which was not certified by peer review) is the author/funder. This article is a US Government work. It is not subject to copyright under 17 USC 105 and is also made available for use under a CC0 license.

Parameters of CRU-based model (agent-based model)

The full set of model parameters and computer code are given in our original publication [36]. The dormant-cell model tested in the present study had the following specific parameters: CRU positions were distributed uniformly at random, and g_{CaL} was 0.27 nS/pF (see Figure 7B in [36]).

Important note about cell membrane capacitance.

SAN pacemaker cell sizes are extremely heterogeneous, with cell membrane capacitance varying from 10 to 100 pF [39,41,79]. Hence, the same current produces a stronger effect in smaller cells, because the V_m change dV during time period dt is directly proportional to the current dI but inversely proportional to the membrane capacitance Cm: dV=- dI*dt/Cm. Our model simulates function of a smaller (but still realistic) SAN cell having a membrane electrical capacitance of 20 pF. We chose such cell in our numerical model simulations because it better reproduces function of so-called "central cell" located in the SAN center where the leading pacemaker site is located. The cells in the center of the SAN are characterized by smaller size vs. peripheral cells [80,81]. A 20 pF cell model has been previously used by other scientists to simulate function of the central cell [82]. Thus, for example, in our 20 pF cell model current 20 pA noise (density 1 pA/pF) would produce similar V_m change as 62.5 pA current in a 62.5 pF cell (same density of 1 pA/pF).

Parameters of Maltsev-Lakatta models (common pool models)

The full set of model parameters is given in our original publication [38]. To generate models representing different cell populations, we varied two key parameters of the coupled-clock system, g_{CaL} and P_{up}. Specifically, the model sensitivity analysis (Figure 8A-D) was performed by varying g_{CaL} from 0.28 to 0.52 nS/pF in 0.0025-nS/pF increments and P_{up} from 0 to 12 mM/s

bioRxiv preprint doi: https://doi.org/10.1101/2024.12.19.629452; this version posted May 4, 2026. The copyright holder for this preprint (which was not certified by peer review) is the author/funder. This article is a US Government work. It is not subject to copyright under 17 USC 105 and is also made available for use under a CC0 license.

in 0.2-mM/s increments. In panels E, F, and G of Figure 8, the fast-firing cell was modeled with g_{CaL} = 0.464 nS/pF and P_{up} = 12 mM/s (the standard Maltsev-Lakatta model), the slow-firing cell with g_{CaL} = 0.43 nS/pF and P_{up} = 1.5 mM/s, and the dormant cell with g_{CaL} = 0.4 nS/pF and P_{up} = 4 mM/s.

The effect of cholinergic receptor stimulation with 0.1 μM acetylcholine was modeled as previously described[59]. In brief, the fractional block (b_{CaL}) of I_{CaL} during cholinergic receptor stimulation was adopted from the model of Zaza et al.[83], as given in the legend to their Figure 2:

$$g_{CaL\_ACh} = C_m \cdot g_{CaL} \cdot (1 - b_{CaL})$$

$$b_{CaL} = [acetylcholine]^{\wedge}0.348/(2921^{\wedge}0.348+[\ acetylcholine]^{\wedge}0.348),$$

where [acetylcholine] is given in μM

Thus, the fractional block in the presence of 0.1 μM acetylcholine (simulated in the present study) was relatively small: $b_{CaL}(0.1)$ = 0.02728, that is, <3%. The shift s (in mV) of the I_f activation curve during cholinergic receptor stimulation was adopted from Zhang et al.[84] as follows:

$$V_{If,1/2\_ACh} = V_{If,1/2} + s$$

$$s = s_{max}[acetylcholine]^{n_f} / (K_{0.5,f}^{n_f} + [acetylcholine]^{n_f})$$

s_{max} = -7.2 mV: maximum acetylcholine-induced shift of I_f half activation voltage.

n_f = 0.69 and $K_{0.5,f}$ = 12.6 nM: Michaelis-Menten parameters for acetylcholine modulation of I_f.

For 100 nM of acetylcholine: s_{max} =-5.81 mV and $V_{If,1/2\_ACh}$ = -64 - 5.81 = - 69.81mV

The formulation of the acetylcholine-activated K current (I_{KACh}) was adopted from Demir et al.[85] (note that I_{KACh} = 0 when [acetylcholine] = 0), with g_{KACh} = 0.14241818 nS/pF.

$$I_{KACh} = a \cdot g_{KACh} \cdot (V_m - E_K)$$

$$beta = 0.001 \cdot 12.32/(1+0.0042/[acetylcholine])\ \text{(per ms)}$$

bioRxiv preprint doi: https://doi.org/10.1101/2024.12.19.629452; this version posted May 4, 2026. The copyright holder for this preprint (which was not certified by peer review) is the author/funder. This article is a US Government work. It is not subject to copyright under 17 USC 105 and is also made available for use under a CC0 license.

$$alfa = 0.001 \cdot 17 \cdot \exp(0.0133 \cdot (V_m + 40)) \text{ (per ms)}$$

$$a_\infty = beta / (alfa + beta)$$

The present study did not investigate I_{KACh} kinetics or system transitions after acetylcholine application. Therefore, in our simulations I_{KACh} was assumed to have reached steady state, with a set to the steady-state value defined above. Thus,

$$I_{KACh} = a_\infty \cdot g_{KACh} \cdot (V_m - E_K)$$

cholinergic receptor stimulation inhibits P_{up}, with fractional block b_{up} formulated as follows:

$$P_{up\_ACh} = P_{up} \cdot (1 - b_{up})$$

$$b_{up} = b_{up,max} \cdot [\text{acetylcholine}]/(K_{0.5,up} + [\text{acetylcholine}])$$

where $K_{0.5,up}$ = 90 nM is the [acetylcholine] for half-maximal inhibition and $b_{up,max}$ = 0.7.

For 100 nM of acetylcholine: b_{up} =0.368421052632.

Parameters of SAN tissue models (2D grid multi-cell model)

The full set of model parameters is given in our original publication [42]. To generate the sinus-arrest model examined here, we distributed tissue-model parameters over the range g_{CaL} = 0.37 nS/pF and $0 < P_{up} < 9.5$ mM/s (magenta double-headed arrow in Figure S12) so that most cells were dormant and the model progressed into sinus arrest after a few cycles over 6 s.

bioRxiv preprint doi: https://doi.org/10.1101/2024.12.19.629452; this version posted May 4, 2026. The copyright holder for this preprint (which was not certified by peer review) is the author/funder. This article is a US Government work. It is not subject to copyright under 17 USC 105 and is also made available for use under a CC0 license.

Supplemental Figures S1-S12

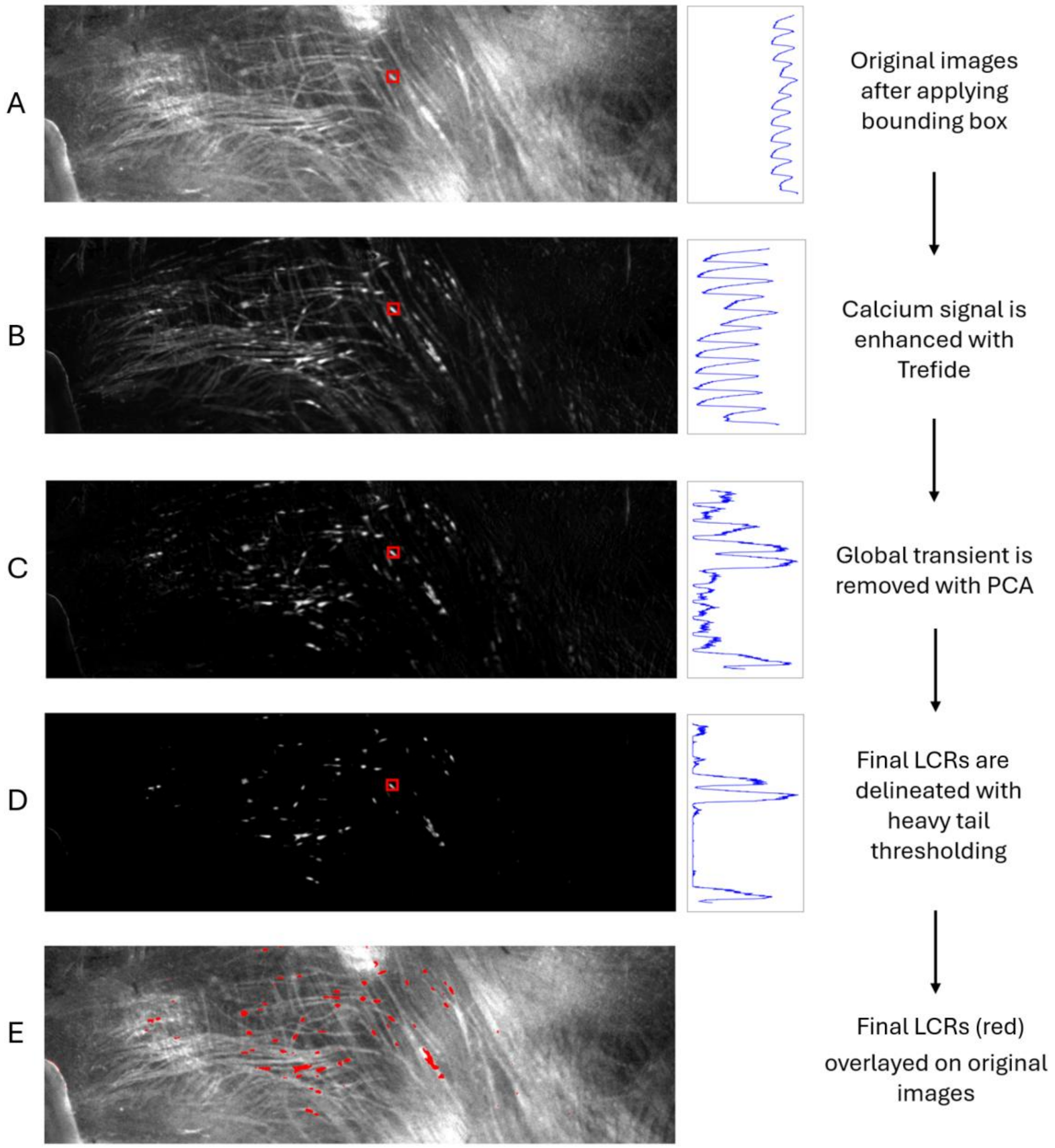

Figure S1. Step-by-step breakdown of the analysis used to identify LCR signals in SAN tissue. **A**, Preprocessed Ca-imaging data following automated tissue-boundary delineation and spatial cropping, showing raw fluorescence intensity after histogram normalization and subthreshold-pixel elimination. **B**, Enhanced spatiotemporal Ca dynamics extracted through Penalized Matrix Decomposition (PMD) via Trefide, revealing synchronized transients, propagating waves, and localized release events within 40 x 40 pixel patches under Total Variation and Trend Filtering constraints. **C**, Residual Ca activity after Principal Component Analysis (PCA) subtraction of the dominant global action-potential-driven wave, preserving intrinsic local signaling while eliminating collective propagation artifacts. **D**, Objective identification of candidate LCRs through EVT-derived thresholding, in which Generalized Pareto Distribution (GPD) fitting across the 50th to 99th percentile range isolates high-amplitude spatiotemporal events in the extreme tail of the distribution. **E**, Composite visualization overlaying EVT-identified LCRs (red) onto the original preprocessed tissue, thereby showing background Ca signals on the original data. Small red boxes in the images in panels A-D outlines the same Region of Interest inside the tissue, with spatially averaged signal shown in the respective right-hand panels as a function of time in parallel with key steps in image processing.

bioRxiv preprint doi: https://doi.org/10.1101/2024.12.19.629452; this version posted May 4, 2026. The copyright holder for this preprint (which was not certified by peer review) is the author/funder. This article is a US Government work. It is not subject to copyright under 17 USC 105 and is also made available for use under a CC0 license.

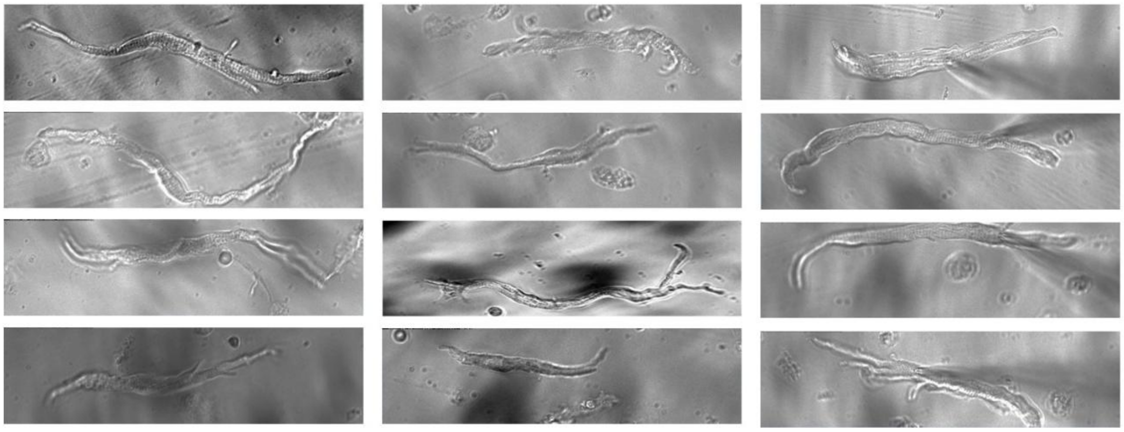

Figure S2. Representative examples of dormant SAN cells. The cells feature classical long curved, spindle-shaped morphology.

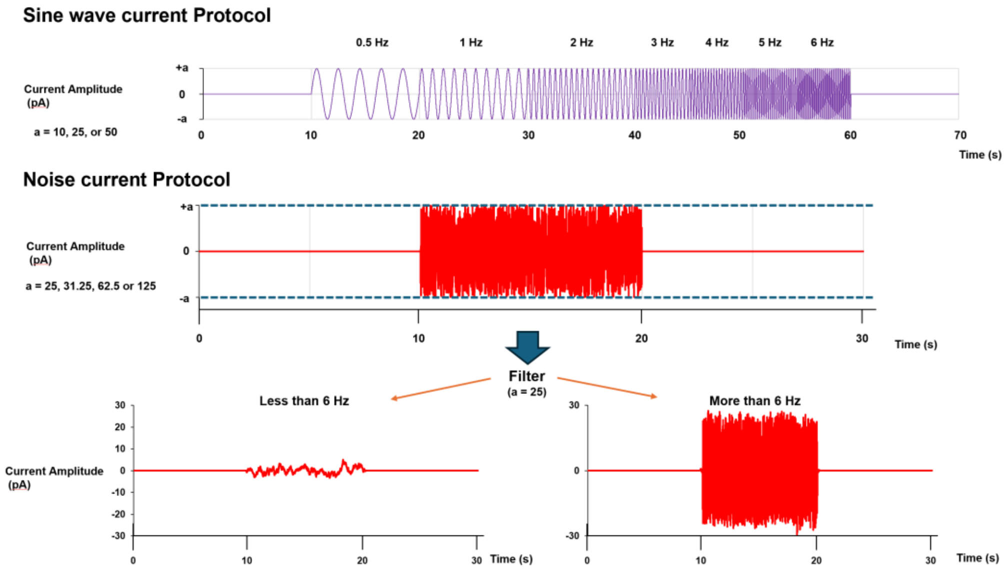

Figure S3. Sine wave and noise current protocols used in the study. The sine wave was applied for 50 s with sine-wave frequency increasing from 0.5 Hz to 6 Hz (upper panel). The current white noise I_{noise} was generated as a sequence of random numbers within a range of [-a, a] with an interval of 4 ms. It is important to note that while the noise amplitudes seem to be substantial, i.e. comparable with those of major currents (like I_f or I_{CaL}), SAN cells, in fact, can process and react with one-to-one capture to only those frequency components (embedded in the white noise) which are within their resonance spectrum, i.e. below 6 Hz (the highest rate of rabbit heart, see also previous

bioRxiv preprint doi: https://doi.org/10.1101/2024.12.19.629452; this version posted May 4, 2026. The copyright holder for this preprint (which was not certified by peer review) is the author/funder. This article is a US Government work. It is not subject to copyright under 17 USC 105 and is also made available for use under a CC0 license.

section). Thus, after a 6 Hz-low-pass filtering, the amplitude of our white noise protocol (processed by cells) decreased approximately by a factor of 5 (left bottom panel). Note, in patch-clamp experiments using Axopatch 200B patch-clamp amplifier (Molecular Devices), application of positive external currents depolarizes the cell membrane, and application of negative external currents hyperpolarizes the cell membrane.

bioRxiv preprint doi: https://doi.org/10.1101/2024.12.19.629452; this version posted May 4, 2026. The copyright holder for this preprint (which was not certified by peer review) is the author/funder. This article is a US Government work. It is not subject to copyright under 17 USC 105 and is also made available for use under a CC0 license.

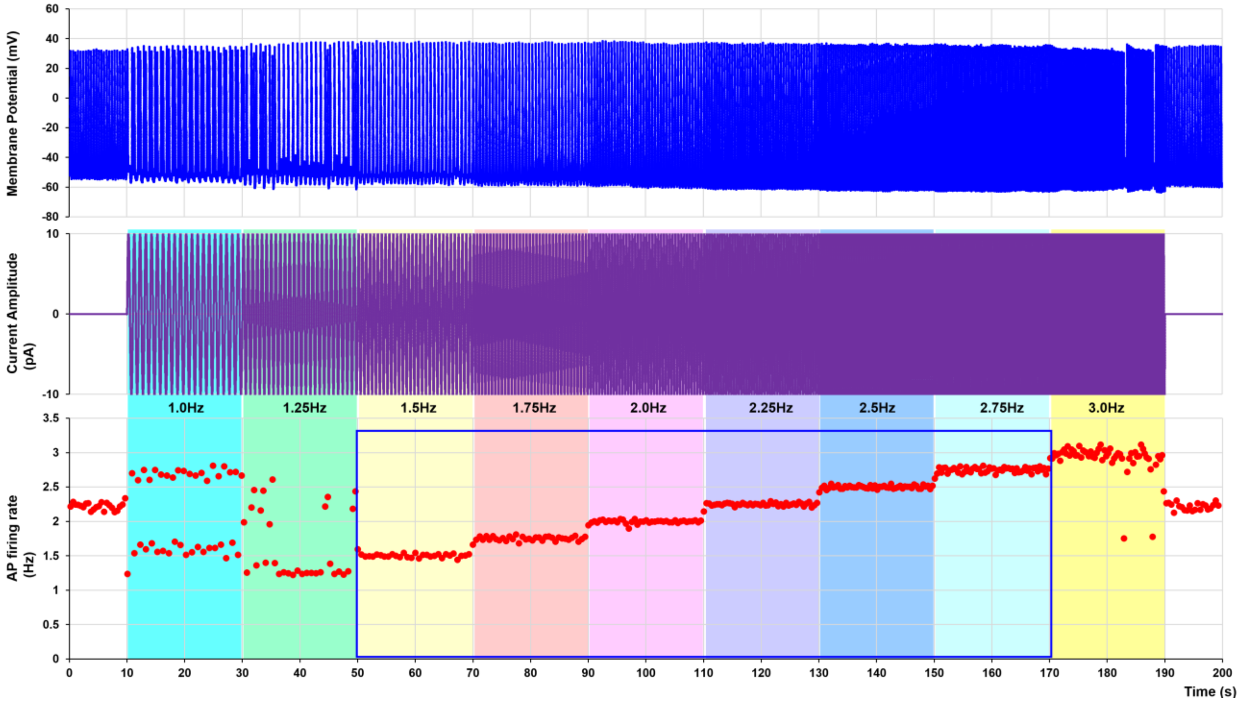

Figure S4. The resonance spectrum is continuous. Shown is an example of one-to-one rhythm capture in a representative cell within its resonance spectrum (from 1.5 to 2.75 Hz) to a sequence of 10 pA sine waves with a small (0.25 Hz) consecutive frequency increase.

bioRxiv preprint doi: https://doi.org/10.1101/2024.12.19.629452; this version posted May 4, 2026. The copyright holder for this preprint (which was not certified by peer review) is the author/funder. This article is a US Government work. It is not subject to copyright under 17 USC 105 and is also made available for use under a CC0 license.

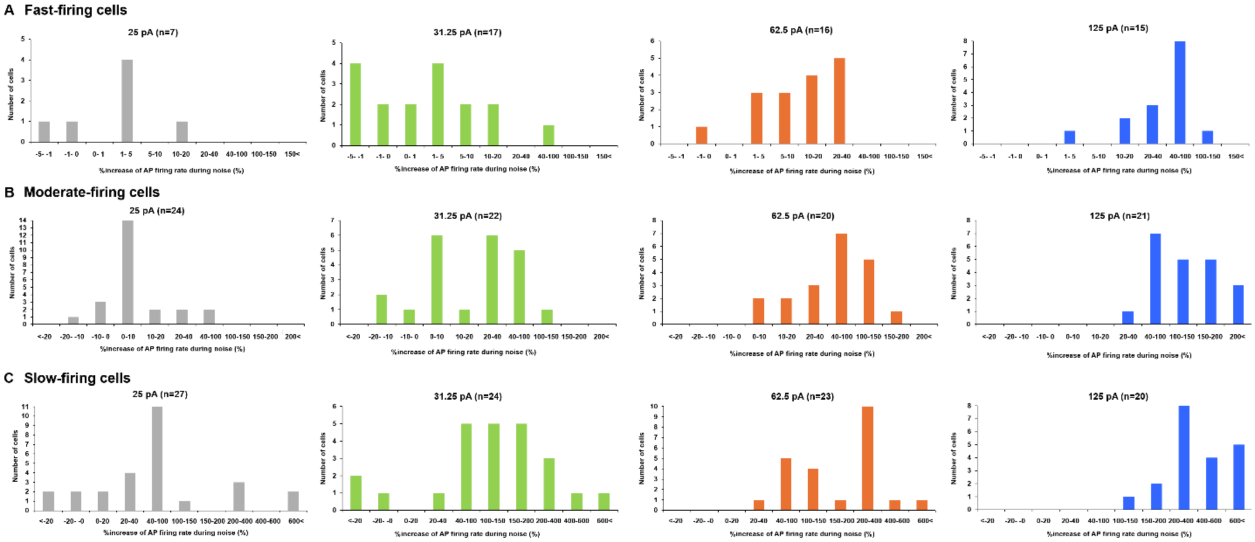

Figure S5. Distribution of the noise effect on the increase in average AP firing rate among all tested cells in each cell population for each noise amplitude. A, In the fast-firing group, all cells increased their average AP firing rate at 125 pA noise amplitude. Almost all cells increased their average AP firing rate at 62.5 pA noise (16/17 cells). At 31.25 pA noise, average firing rate increased in 11/17 cells. At 25 pA noise, it increased in 5/7 cells. **B**, In the moderate-firing group, all cells increased their average AP firing rate at 62.5 and 125 pA noise. At 25 or 31.25 pA noise, average firing rate increased in 20/24 or 19/22 cells, respectively. **C**, In the slow-firing group, all cells increased their average AP firing rate at 62.5 and 125 pA noise. At 25 or 31.25 pA noise, average firing rate increased in 23/27 or 21/24 cells, respectively.

bioRxiv preprint doi: https://doi.org/10.1101/2024.12.19.629452; this version posted May 4, 2026. The copyright holder for this preprint (which was not certified by peer review) is the author/funder. This article is a US Government work. It is not subject to copyright under 17 USC 105 and is also made available for use under a CC0 license.

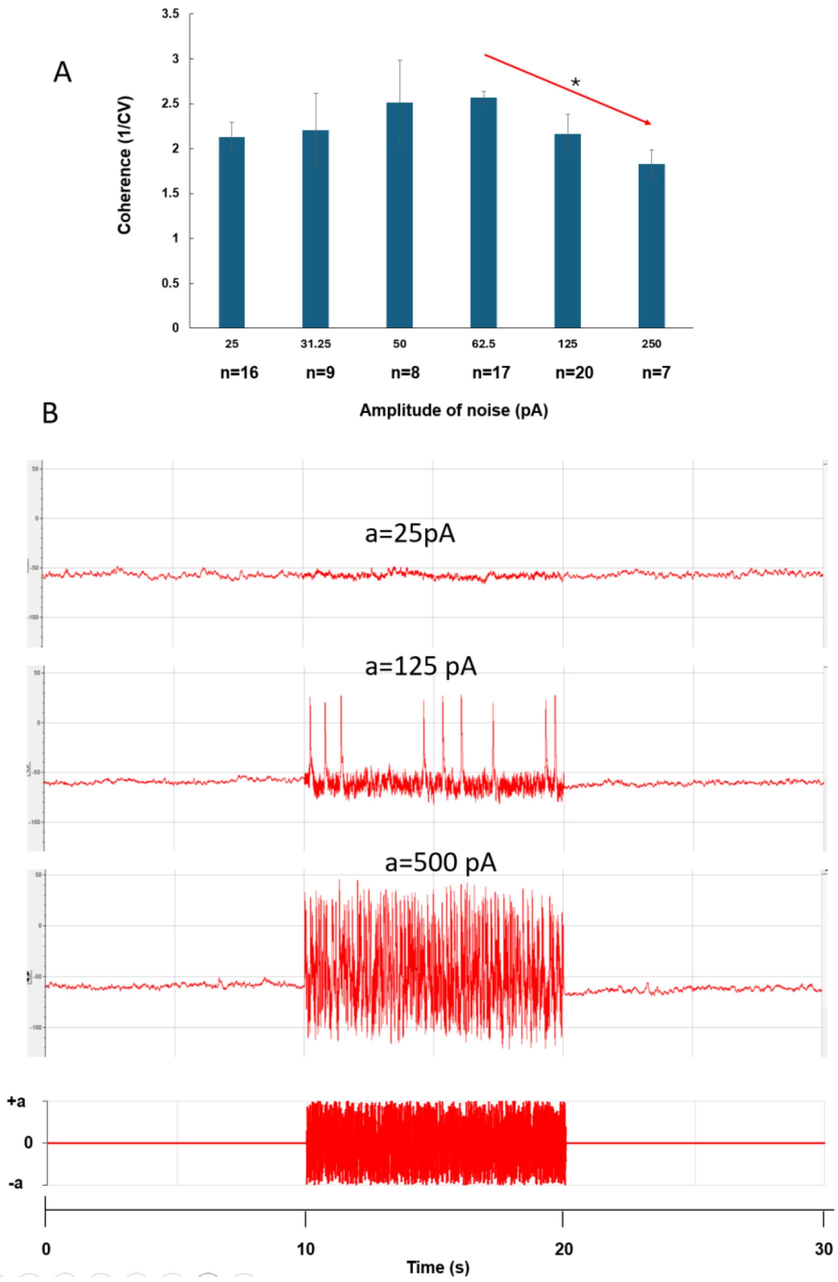

Figure S6. Optimal noise amplitude in stochastic resonance in a dormant cell. **A**, Temporal coherence, defined as the inverse of the coefficient of variation, revealed the classic bell-shaped profile of stochastic resonance, with firing rhythmicity being optimal at moderate noise amplitudes but low at very small and very large noise amplitudes. The Jonckheere-Terpstra trend test revealed decreasing trends in 1/CV between 62.5 pA and 250 pA (arrows; *P<0.05). **B**, Examples of original consecutive V_m recordings (in same cell) over a wide range of noise amplitudes, including extremely small and extremely strong noise. With extremely strong noise (bottom subpanel), V_m exhibited high-amplitude fluctuations deviating almost equally up and down from the baseline. AP upstrokes could not be resolved, and AP fidelity was lost.

bioRxiv preprint doi: https://doi.org/10.1101/2024.12.19.629452; this version posted May 4, 2026. The copyright holder for this preprint (which was not certified by peer review) is the author/funder. This article is a US Government work. It is not subject to copyright under 17 USC 105 and is also made available for use under a CC0 license.

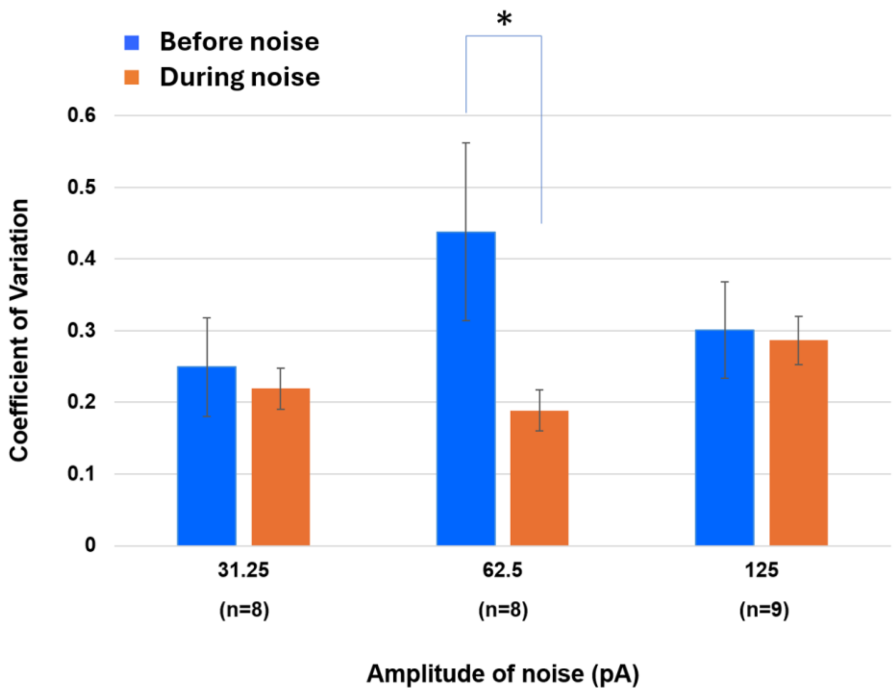

Figure S7. Analysis of coefficient of variation (SD/mean) of the firing frequency before and during noise of different amplitudes in cells that became slow-firing under carbachol. Substantial and statistically significant (*$p < 0.05$, paired t-test) decrease in CV was found only at an intermediate (optimal) noise amplitude of 62.5 pA. The decrease in CV at lower and higher noise amplitudes was small and not significant.

bioRxiv preprint doi: https://doi.org/10.1101/2024.12.19.629452; this version posted May 4, 2026. The copyright holder for this preprint (which was not certified by peer review) is the author/funder. This article is a US Government work. It is not subject to copyright under 17 USC 105 and is also made available for use under a CC0 license.

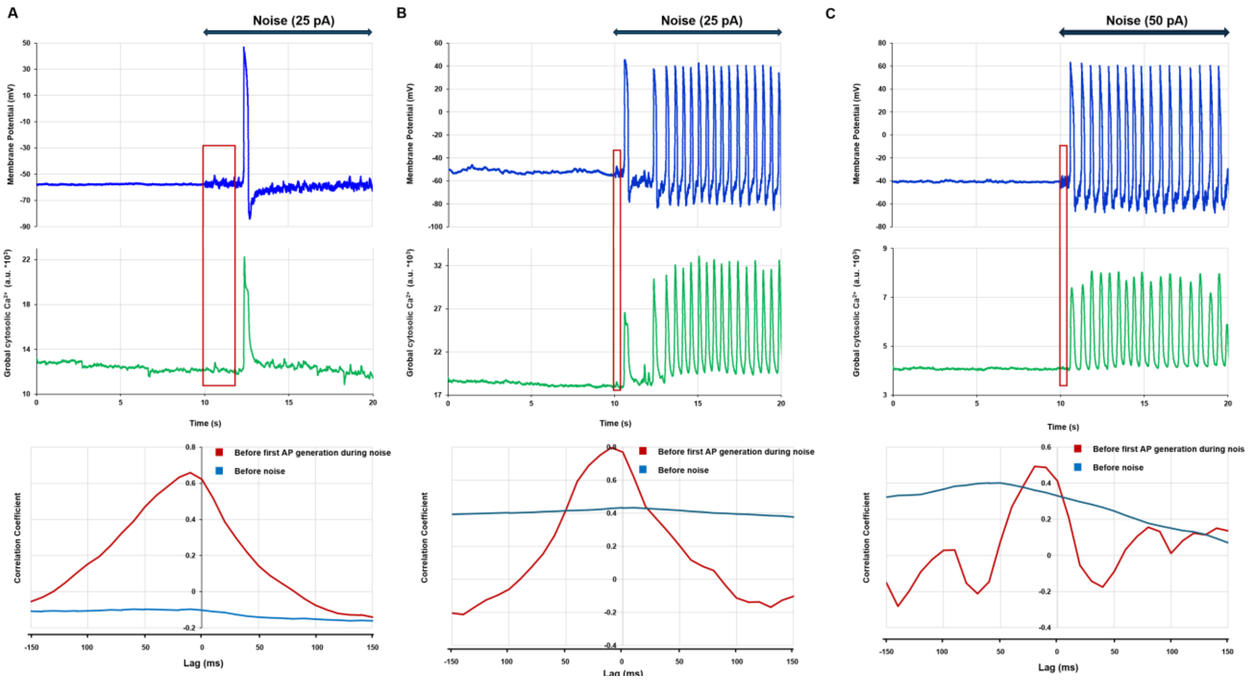

Figure S8. Stochastic resonance is amplified by the coupled-clock system in SAN cells. A-C, Representative examples of stochastic resonance in dormant cells during simultaneous perforated-patch recordings of V_m and Ca signals (top panels) and the corresponding cross-correlation functions of V_m and Ca for subthreshold signaling before and during noise application (bottom panels). The time windows selected for cross-correlation analysis (before the first AP fired) are indicated by the squares. Cross-correlation of subthreshold signals increased substantially during noise application, culminating in AP generation via stochastic resonance.

bioRxiv preprint doi: https://doi.org/10.1101/2024.12.19.629452; this version posted May 4, 2026. The copyright holder for this preprint (which was not certified by peer review) is the author/funder. This article is a US Government work. It is not subject to copyright under 17 USC 105 and is also made available for use under a CC0 license.

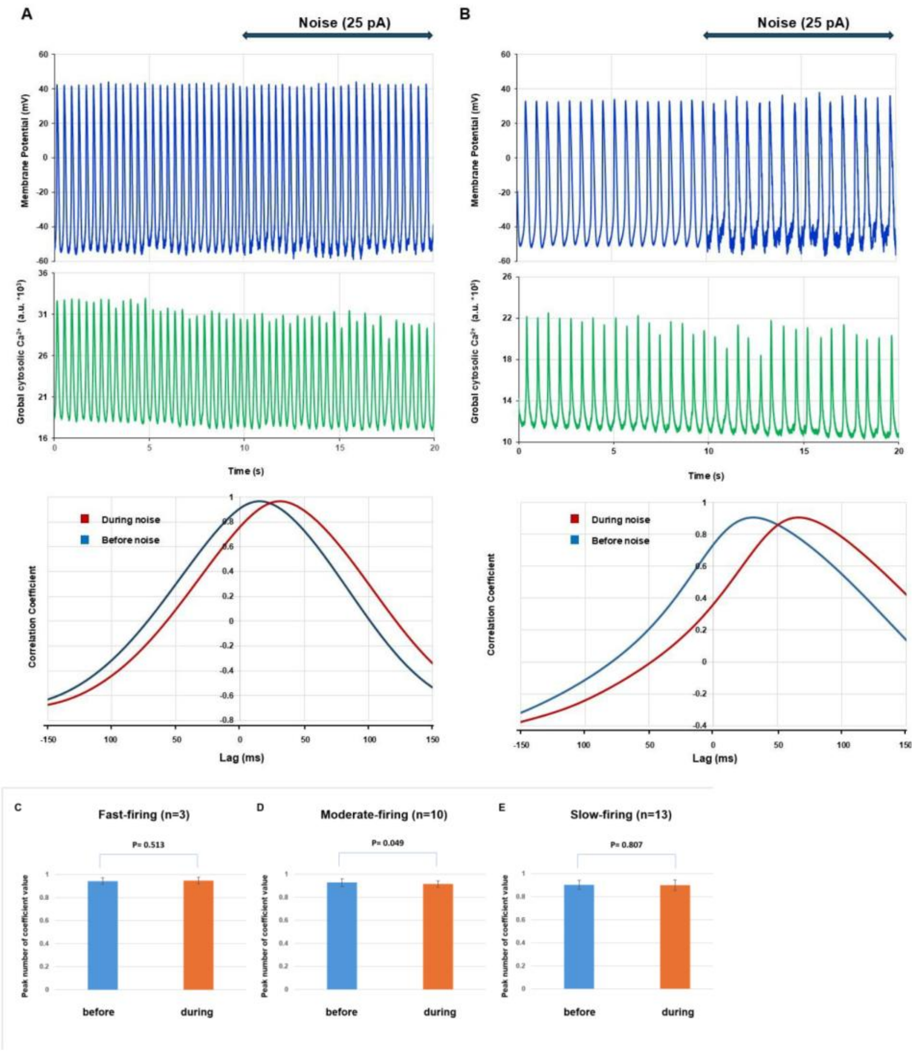

Figure S9. Coupling of V_m and Ca remains strong in the presence of noise in AP-firing-cells independent of their firing frequency. A and B: Representative examples of simultaneous perforated patch recordings of V_m and Ca signals (top panels) in AP firing cells in absence or presence of noise and their respective cross-correlation functions of V_m and Ca showing strong V_m-Ca coupling independent of presence of noise. **C-E**: Statistical analysis showed no statistically significant change in peak value of cross-correlation in the presence of noise in all firing cell groups.

bioRxiv preprint doi: https://doi.org/10.1101/2024.12.19.629452; this version posted May 4, 2026. The copyright holder for this preprint (which was not certified by peer review) is the author/funder. This article is a US Government work. It is not subject to copyright under 17 USC 105 and is also made available for use under a CC0 license.

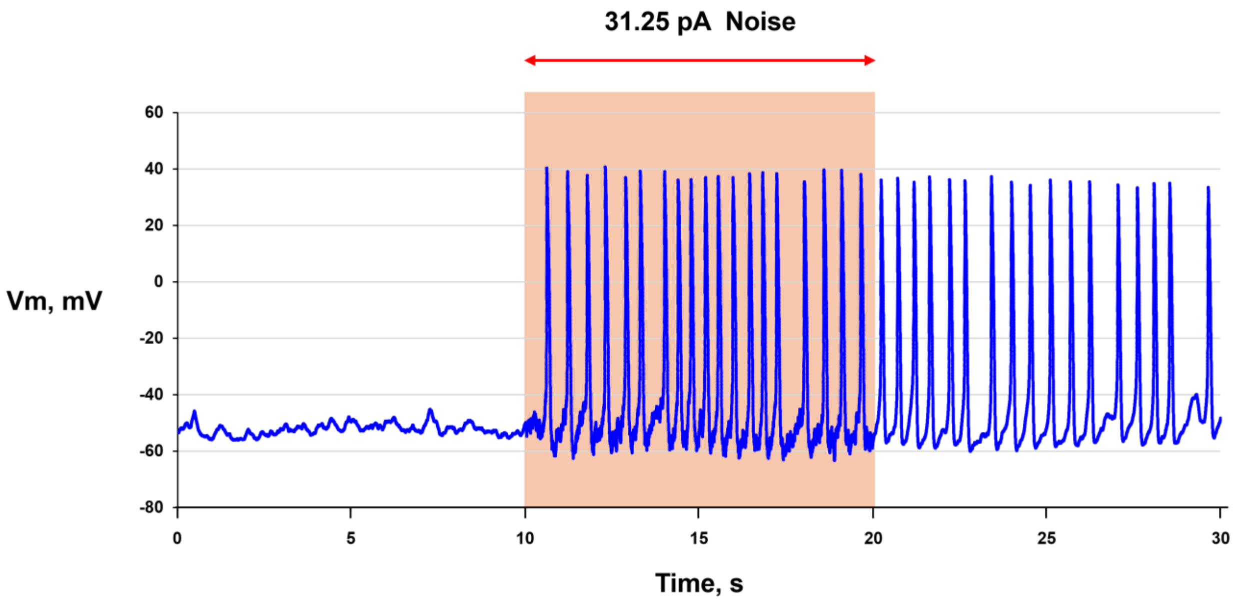

Figure S10. Example of a memory effect in a dormant SAN cell. Stochastic resonance awakened the cell to fire APs that continued in the absence of noise after 10 s of noise application.

bioRxiv preprint doi: https://doi.org/10.1101/2024.12.19.629452; this version posted May 4, 2026. The copyright holder for this preprint (which was not certified by peer review) is the author/funder. This article is a US Government work. It is not subject to copyright under 17 USC 105 and is also made available for use under a CC0 license.

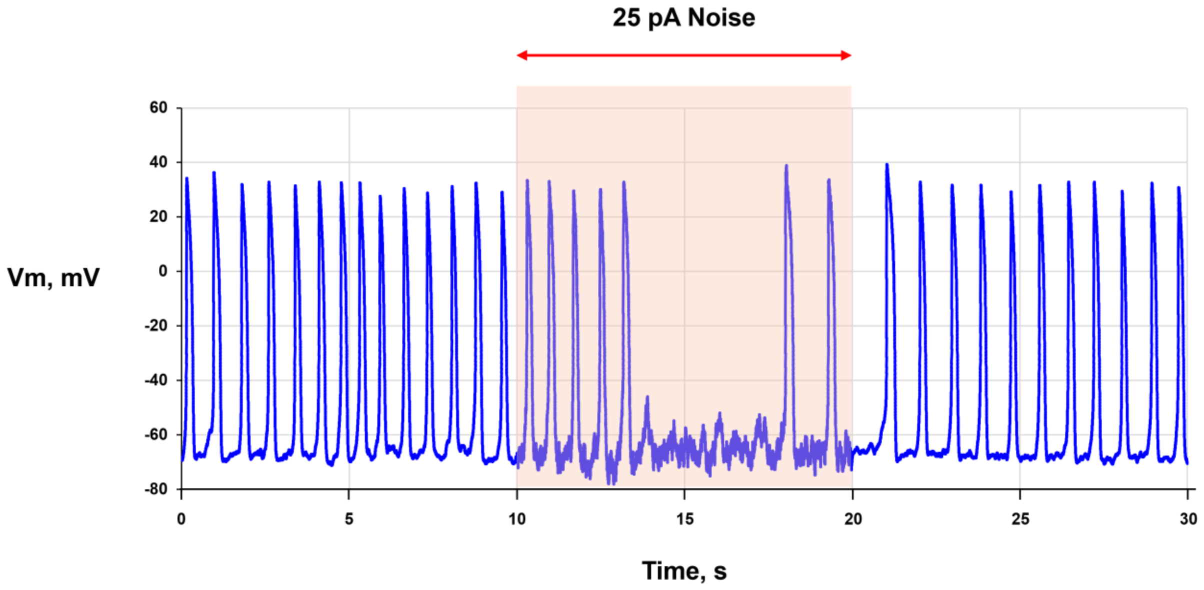

Figure S11. Example of inverse stochastic resonance in a SAN cell. Noise suppressed AP firing in the cell, but AP firing resumed after noise removal.

bioRxiv preprint doi: https://doi.org/10.1101/2024.12.19.629452; this version posted May 4, 2026. The copyright holder for this preprint (which was not certified by peer review) is the author/funder. This article is a US Government work. It is not subject to copyright under 17 USC 105 and is also made available for use under a CC0 license.

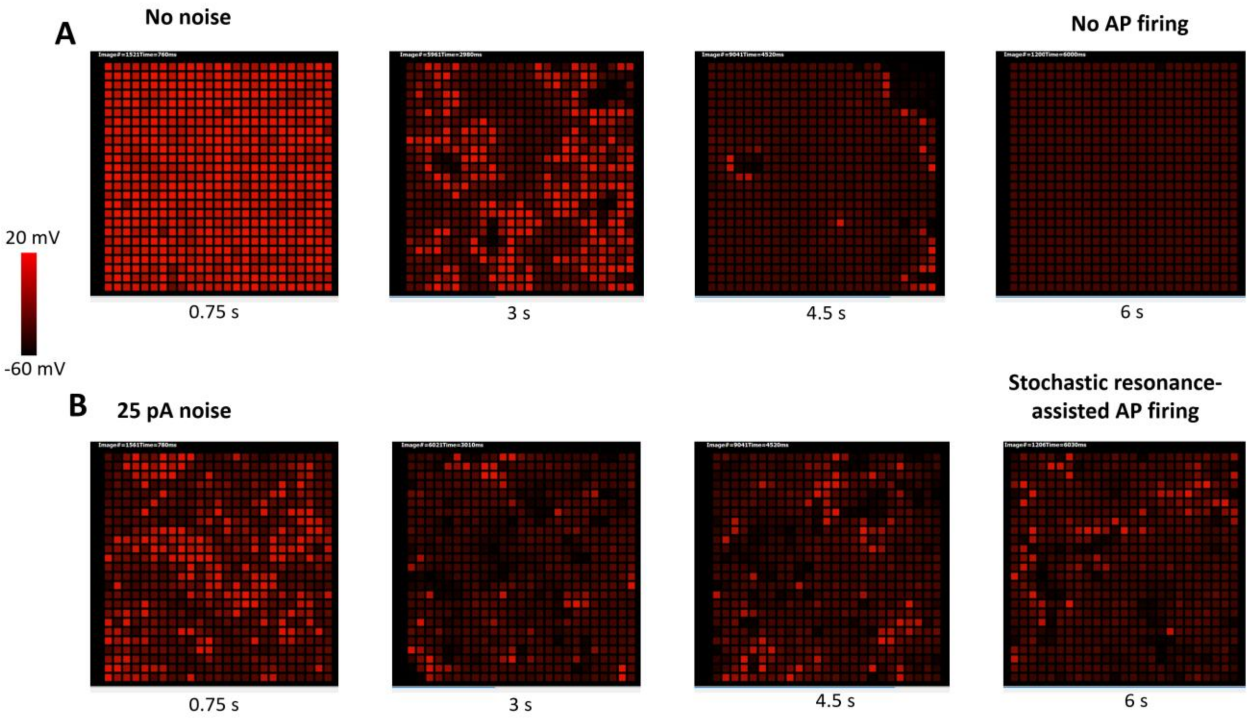

Figure S12. Stochastic resonance ensures fail-safe SAN operation bordering sinus arrest in our numerical simulations of SAN tissue model. Shown is an example of noise effect in a simulation of a heterogeneous SAN tissue model (25x25 cells) with P_{up} uniformly randomly distributed within the range [0, 9.5] mM/s and fixed g_{CaL}=0.37 nS/pF. The model progressed into sinus arrest in the absence of noise, but continued AP firing in the presence of noise. Each subpanel shows instant distribution of V_m by red shades (<−60 mV as pure black to > 20 mV as pure red). See also Video S10 for V_m dynamics in the tissue model simulations.

bioRxiv preprint doi: https://doi.org/10.1101/2024.12.19.629452; this version posted May 4, 2026. The copyright holder for this preprint (which was not certified by peer review) is the author/funder. This article is a US Government work. It is not subject to copyright under 17 USC 105 and is also made available for use under a CC0 license.

Cell Number	Noise Level (pA)	L-Kurtosis Before Noise	L-Kurtosis with Noise
1	12.5	0.4414	0.5716
2	25	0.5466	0.5589
3	50	0.6367	0.7194
4	62.5	0.6159	0.6875
5	50	0.5013	0.6381
Average		0.548*	0.635*

Table S1. Quantitative assessment of noise-induced distributional changes using L-kurtosis, the ratio of the fourth to the second L-moment of a statistical distribution. The table shows L-kurtosis ($\tau 4$) before and during application of different noise levels to five dormant SAN cells. Higher L-kurtosis indicates a distribution with heavier tails (more outliers), whereas lower L-kurtosis indicates lighter tails (fewer outliers). All five analyzed cells showed increased L-kurtosis following noise exposure, indicating enhanced tail weight and relative enrichment of large-scale LCR events. One-tailed paired t-tests confirmed this shift toward heavier-tailed distributions ($p<0.01$, Cohen's $d = 1.719$), supporting the conclusion that noise modulates LCR-event architecture.

bioRxiv preprint doi: https://doi.org/10.1101/2024.12.19.629452; this version posted May 4, 2026. The copyright holder for this preprint (which was not certified by peer review) is the author/funder. This article is a US Government work. It is not subject to copyright under 17 USC 105 and is also made available for use under a CC0 license.

Legends for Videos

Videos S1-S10

Video S1. **Presence of biological noise in intact SAN tissue.** **Top:** Low-zoom microscopic view of the entire mouse SAN preparation loaded with the Ca indicator Fluo-4, illustrating AP-induced Ca transients (APCTs) imaged by a high-speed camera. Anatomical benchmarks: crista terminalis, superior vena cava (SVC), hepatic vein (HV), and inferior vena cava (IVC). **Middle and bottom panels**: Higher-magnification views of a region of interest within the central SAN area. Local Ca release (LCR) signals detected by our novel algorithm are shown in pink.

Video S2. **Presence of biological noise in intact SAN tissue.** **Top:** Another high-magnification video of Ca signals in a region of interest within the central SAN area. **Bottom**: Local Ca release (LCR) signals detected by our novel algorithm are shown in pink, overlaid on the original video.

Video S3. **Examples of slow Ca waves in two SAN tissues.** Red boxes outline the locations of the slow Ca waves (low-frequency LCRs). The upper video was recorded at a sampling rate of 502.091 fps until frame #1284 (total duration 2.557 s). The lower video was recorded at 656.581 fps until frame #1736 (total duration 2.643 s).

Video S4. **Examples of multiple smaller and faster LCR signals occurring within individual cells.** Green boxes outline the locations of the LCR signals. The upper video was recorded at a

bioRxiv preprint doi: https://doi.org/10.1101/2024.12.19.629452; this version posted May 4, 2026. The copyright holder for this preprint (which was not certified by peer review) is the author/funder. This article is a US Government work. It is not subject to copyright under 17 USC 105 and is also made available for use under a CC0 license.

sampling rate of 30 fps until frame #160 (total duration 5.3 s). The lower video was recorded at 613.333 fps until frame #1100 (total duration 1.793 s).

Video S5. Examples of incoherent APCT firing in individual cells. Blue boxes outline the locations of the incoherent firing signals. The upper video was recorded at a sampling rate of 644.192 fps until frame #1100 (total duration 1.707 s). The lower video was recorded at 656.581 fps until frame #1727 (total duration 2.630 s).

Video S6. Examples of three patterns of background Ca signals detected in individual cells by confocal microscopy. Red circles indicate the locations of the signals. The blue box indicates a zoomed view illustrating small, frequent LCRs.

Video S7. Example simulation of a CRU-agent model of a dormant SAN cell awakened to fire APs by application of white noise. Spatiotemporal synchronization (black arrows) of subthreshold Ca oscillations (in green, middle panel) in the presence of noise (in grey, bottom panel). The top panel shows local Ca dynamics in red shades (10 μM = pure red; <0.15 μM = pure black) and CRUs in different functional states: ready to fire, green; refractory, blue; and releasing Ca, shades of gray reflecting junctional sarcoplasmic reticulum [Ca] dynamics (>0.3 mM = white; 0 = black). This clip is part of a longer simulation and shows 2 s before and 2 s after noise onset (outlined by a box in Figure 7A).

bioRxiv preprint doi: https://doi.org/10.1101/2024.12.19.629452; this version posted May 4, 2026. The copyright holder for this preprint (which was not certified by peer review) is the author/funder. This article is a US Government work. It is not subject to copyright under 17 USC 105 and is also made available for use under a CC0 license.

Video S8. Importance of Ca-V_m coupling for stochastic resonance. Representative example of stochastic resonance in a dormant cell obtained during simultaneous experimental recordings of Ca signals and V_m, with videos of Ca signals shown before noise and during noise application (right panels). Noise increased local Ca-release activity coupled to V_m changes before the first AP and its associated APCT were generated (time window indicated by the yellow box in the top left panel). The increase in coupling is reflected by the higher peak of the V_m-Ca cross-correlation function (left, bottom panel).

Video S9. White-noise current substantially expanded the parametric space of AP firing in single-cell models (each cell in the grid represents a separate cell model). Application of white noise in the models simulating single SAN cell function (right panels versus left panels) expanded AP firing toward where cells failed to operate without noise, both in the basal state (upper panels) and during cholinergic receptor stimulation (bottom panels). Each movie panel represents the result of numerical simulations of 5,917 (97 by 61 grid in xy) models with different coupled-clock parameters. I_{CaL} conductance (g_{CaL}) was distributed evenly along the x-axis from 0.28 to 0.52 nS/pF in 0.0025-nS/pF increments. The sarcoplasmic reticulum Ca pumping rate (P_{up}) was distributed evenly along the y-axis from 0 to 12 mM/s in 0.2-mM/s increments. White noise of 25 pA amplitude was added to each individual (not connected) SAN cell model in the grid. V_m dynamics in each model are coded by red shades from black (<-60 mV) to pure red (>20 mV). Image number and simulation time are shown in the upper left corner of each video panel. See also Figure 8A-D.

bioRxiv preprint doi: https://doi.org/10.1101/2024.12.19.629452; this version posted May 4, 2026. The copyright holder for this preprint (which was not certified by peer review) is the author/funder. This article is a US Government work. It is not subject to copyright under 17 USC 105 and is also made available for use under a CC0 license.

Video S10. **Example of the effect of noise in simulations of the SAN tissue model**. The SAN model progressed into sinus arrest after a few synchronized cycles in the absence of noise (left panel), whereas in the presence of noise the SAN model continued AP firing (right panel). Each cell in the square grid of the SAN model is represented by a box colored in red shades corresponding to V_m from <-60 mV (pure black) to >20 mV (pure red).

Glossary of Key Terms

Sinoatrial node (SAN): A small region of specialized cardiac tissue in the right atrium that spontaneously generates APs to pace the heartbeat.

Dormant cell: A SAN cell that generates subthreshold oscillations but does not produce APs under basal conditions; can be awakened by adrenergic stimulation or noise.

Stochastic resonance: A phenomenon in which the addition of an optimal level of noise enables a subthreshold signal to reach detection threshold, improving system output.

Inverse stochastic resonance: A phenomenon in which noise suppresses rather than enhances oscillatory activity, observed here in some fast-firing cells.

Subthreshold signal: A signal whose amplitude is insufficient to independently trigger an AP.

Resonance spectrum: The range of external signal frequencies to which a given SAN cell responds with one-to-one AP capture; equivalent to the cell's functional frequency processing range.

Coupled-clock system: The interaction between membrane ion channel oscillations (membrane clock) and intracellular Ca cycling (Ca clock) that together drive rhythmic AP generation in SAN cells.

bioRxiv preprint doi: https://doi.org/10.1101/2024.12.19.629452; this version posted May 4, 2026. The copyright holder for this preprint (which was not certified by peer review) is the author/funder. This article is a US Government work. It is not subject to copyright under 17 USC 105 and is also made available for use under a CC0 license.

Local Ca release (LCR): A spontaneous, localized intracellular Ca release event generated by ryanodine receptor channels in the sarcoplasmic reticulum during diastolic depolarization.

White noise: A random signal with equal power at all frequencies, used here to simulate the broadband biological noise present in SAN tissue.

Coefficient of variation (CV): The ratio of standard deviation to mean of AP firing frequency, used as a measure of firing rhythmicity; lower CV indicates more regular firing.

AP-induced Ca transients (APCTs): periodic synchronized flashes of synchronized Ca releases throughout the SAN tissue induced by respective action potential firing via classical Ca-induced Ca-release (CICR) mechanism.

Ca-induced Ca-release (CICR): Ca release that is triggered by Ca entered via L-type Ca channels (classical CICR) or by a local neighboring Ca release, resulting in propagating Ca release in the form of Ca wave that, in turn, could be short abrupted wave (wavelet or LCR) or full-cell wave (global Ca wave).

References: see reference list in the main text
